# Time-restricted feeding reverses kidney fibrosis through reducing CD8^+^ T cell infiltration in obese mice

**DOI:** 10.1172/jci.insight.195335

**Published:** 2026-08-20

**Authors:** Claudia J. Edell, John D. Erickson, Xiaofen Liu, Savannah C. Walker, Jackson Colson, Michael Heim, Pranav Nagila, Kyle H. Moore, Keri M. Kemp, Kelly Hyndman, Selene Meza-Perez, Troy D. Randall, Annye P. Bennett, Anna G. Sorace, Yu-Hua Dean Fang, David M. Pollock, Carmen De Miguel, Julienne L. Carstens, Jennifer S. Pollock

**Affiliations:** 1 Cardio-Renal Physiology & Medicine,; 2 Division of Nephrology, Department of Medicine,; 3 Division of Clinical Immunology & Rheumatology, Department of Medicine,; 4 Department of Radiology,; 5 Department of Neurology, and; 6 Department of Hematology and Oncology, Heersink School of Medicine, University of Alabama at Birmingham (UAB), Birmingham, Alabama, USA.

**Keywords:** Inflammation, Nephrology, Obesity, T cells

## Abstract

Obesity is a major risk factor for chronic kidney disease. Time-restricted feeding (TRF) shows promise to reduce kidney inflammation in chronic kidney disease. We hypothesized that TRF blunts kidney fibrosis in obese mice by mitigating T cell inflammation. We used a diet-induced obese mouse model fed a high-fat diet (DIO; 45% fat) ad libitum for 18 weeks followed by 2 weeks of TRF or ad libitum high-fat feeding. We found that TRF reversed kidney fibrosis as well as reduced kidney CD8^+^ T cells in DIO mice. Our study also reveals that DIO mice had increased kidney CD8^+^ T cell infiltration from the small intestine that was blunted with TRF. Furthermore, anti-CD8 intervention in DIO showed reduced kidney fibrosis and damage compared with anti-IgG–treated DIO mice. Single-cell RNA-seq data reveal that DIO increased, while TRF reduced, the frequency of a specific cluster of CD8^+^ T cells that featured high expression of exhaustion/activation genes. Spatial analyses showed DIO mice had significant infiltration of PD-1^+^CD8^+^ T cells near CD31^+^ endothelial cells that was diminished by TRF. In conclusion, this study shows that TRF reversed kidney fibrosis through reducing CD8^+^ T cell infiltration in obese mice.

## Abstract 2

Time-restricted feeding helps protect kidneys in obese mice by reducing harmful immune cells (CD8+ T cells) that cause kidney damage and inflammation.

## Graphical abstract

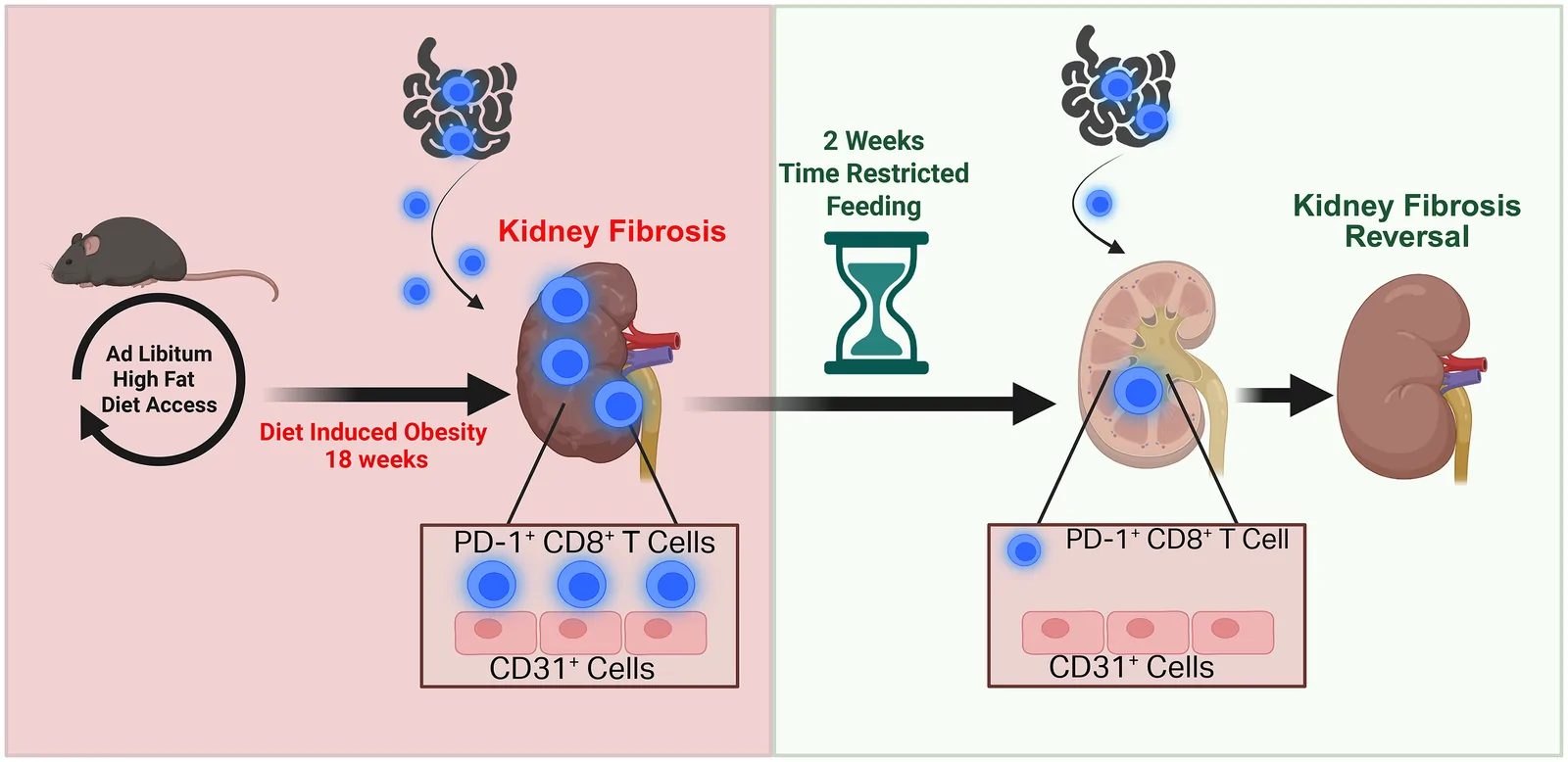

## Introduction

With increasing knowledge of the importance of timing of food intake, time-restricted feeding (TRF) has become a widely popular dietary intervention. TRF limits the timing of when food is consumed but not the type of food or the calories consumed. In obese people, reports show that TRF improved cardiometabolic health (1, 2). We reported that a short-term TRF intervention in obese mice significantly diminished kidney medullary interstitial fibrosis and reduced CD3^+^ T cell numbers compared with obese mice with ad libitum food access (3). However, mechanistic insight of how TRF reduced kidney fibrosis in mice with diet-induced obesity (DIO) was lacking.

There are 2 main subsets of T cells: CD8^+^ and CD4^+^ T cells. Both T cell subsets may be beneficial or harmful to overall health depending on the context. Several investigations report that CD4^+^ and/or CD8^+^ T cells mediate kidney damage in various disease states, including hypertension (4–7), autoimmunity (8, 9), and acute kidney injury (10). However, there is a paucity of data characterizing the immune cell phenotypes within the kidney in obesity.

Immune cells circulate in the blood on a diurnal rhythm, with lymphocyte populations peaking during the end of the sleep phase (11). Immune cell infiltration follows a diurnal rhythm peaking at a distinct time of day for each tissue regulated by endothelial-specific adhesion molecules (12). Within the adipose tissue (AT), obesity-driven changes in chemokine and cytokine production also mediate infiltration of immune cells (13, 14), therefore suggesting distinct differences in immune cell migration patterns in obesity.

Pathogenic T cell infiltration into the kidney is dependent on CC chemokine receptor 5 (CCR5) and CC chemokine ligand 5 (CCL5) (15, 16). Blocking these pathways revealed significantly reduced kidney damage in acute kidney injury and kidney transplant (15, 17). Krebs et al. show in an autoimmune model of crescentic glomerulonephritis that kidney-infiltrating T cells originate from the small intestine (18). Our laboratory reported that T cells mobilize from the small intestine and the draining lymph nodes in response to high salt diet (19). The small intestine immune cell landscape is highly responsive to dietary changes (20–23). Yet, there is little information about T cell migration from the small intestine in response to high-fat diets.

We hypothesized that TRF reduces kidney fibrosis through diminishing T cell infiltration in mice with established DIO. We used a mouse model of DIO assessing changes in kidney fibrosis and damage, diurnal changes in T cell immunolocalization, and immunophenotyping prior to and after a short-term TRF intervention. We also utilized the Kikume Green-Red (KikGR) mouse model (24) to analyze whether immune cell migration from the small intestine and associated tissues infiltrate the kidney and is affected by DIO and TRF. We employed anti-CD8 intervention to identify the T cell subset mediating kidney fibrosis. Finally, we exploited single-cell RNA-seq (scRNA-seq) and multiplex immunofluorescent spatial bioinformatic analysis to identify and localize specific CD8^+^ T cell populations within kidneys of DIO and TRF mice. Collectively, this study reveals that TRF reversed kidney fibrosis in DIO through reducing CD8^+^ T cell migration from the small intestine or associated tissues and infiltration into the kidney. These data highlight the importance of time of day as a biological variable as well as the timing of food intake in obesity on kidney CD8^+^ T cell infiltration mediating kidney fibrosis.

## Results

### TRF reverses kidney interstitial fibrosis in DIO mice.

C57BL6/J mice were placed on high-fat diet (HFD) ad libitum for 18 weeks. Mice continued with ad libitum feeding on HFD (DIO) for 2 additional weeks or 2 weeks of TRF intervention on HFD (DIO+TRF). Mice were also on normal-fat diet (ND) ad libitum for 20 weeks (Figure 1A). DIO and DIO+TRF mice have significantly higher body weight than ND mice (Figure 1B). Two weeks of TRF administered in weeks 18–20 did not result in body weight differences between DIO and DIO+TRF mice (Figure 1B) or in total daily food intake in grams (Supplemental Figure 1A; supplemental material available online with this article; https://doi.org/10.1172/jci.insight.195335DS1). DIO and DIO+TRF mice consumed greater daily kilocalories (kcal) than ND mice with no change in daily kcal intake between DIO and DIO+TRF groups (Figure 1C). Kidney weights were similar between all 3 groups (Figure 1D).

Kidney fibrosis was assessed using a quantitative MRI approach of the apparent diffusion coefficient (ADC). ADC is a noninvasive quantitative assessment that measures the diffusion of water molecules within tissues where free diffusion is reflected by higher ADC and restricted diffusion is reflected by lower ADC. Restricted diffusion occurs through increased fibrosis and inflammation. ADC analyses were completed in the whole kidney after 18 weeks of ND and DIO ad libitum feeding as well as after 20 weeks of DIO ad libitum or TRF intervention in a paired design with the same set of mice. Representative images from the MRI are provided (Supplemental Figure 1B). We found that ADC was lower in DIO mice compared with ND mice after 18 weeks of ad libitum feeding (Figure 1E). Continuing ad libitum feeding to 20 weeks in ND and DIO groups also showed lower ADC in DIO mice compared with ND mice (Figure 1E). Short-term TRF in the DIO group significantly increased ADC (Figure 1E). TRF significantly increased the change in ADC compared with ad libitum feeding in ND and DIO mice, indicating that TRF reverses fibrosis and inflammation in DIO mice (Figure 1E).

We further evaluated kidney medullary and cortical fibrosis via histological analysis of ND and DIO mice at week 18 (pre-TRF) and week 20 (post-TRF) to corroborate the MRI results. Week 18 DIO mice showed fibrosis associated with the medullary vasa recta significantly higher than ND mice (Figure 1, F and G). At week 20, DIO mice also showed increased medullary interstitial fibrosis compared with ND that was significantly reduced by TRF (Figure 1, H and I). In the cortex at week 18, DIO significantly increased cortical tubulointerstitial fibrosis and glomerulosclerosis (Figure 1, J–L) as well as increased the presence of tubular vacuoles (Supplemental Figure 1C) compared with ND. At week 20, TRF significantly reduced cortical tubulointerstitial fibrosis, but not glomerulosclerosis or tubular vacuoles, compared with DIO (Figure 1, M–O, and Supplemental Figure 1D). With picrosirius red staining, the percentage of collagen 1 and 3 was increased in the cortex of DIO mice and reduced with TRF (Supplemental Figure 1E), although longer-term high-fat feeding studies may be necessary for a more robust phenotype in the kidney cortex. TRF also rescued the proximal tubular brush border integrity compared with DIO (Supplemental Figure 1F). These histological assessments at week 18 and week 20 of high-fat feeding confirm the MRI results and demonstrate that TRF reverses kidney medullary and cortical interstitial fibrosis in DIO mice.

### TRF reduces kidney CD8^+^ T cells in the dark phase only of DIO mice.

Previous investigations have found T cells to mediate kidney fibrosis and damage in various models of kidney disease (7–10, 25, 26); however, very few have evaluated T cells in obesity. T cell responses are known to be time-of-day dependent (27, 28); thus, we assessed immunolocalization of CD3^+^ cells (total T cells) within the kidney at both light phase at Zeitgeber Time 1–3 (ZT1–3) and dark phase at ZT13–15. Representative images from the outer medulla and the cortex (Figure 2, A and B) stained for CD3^+^ cells at 2 times of day are shown. DIO increased the number of vasa recta-associated CD3^+^ cells, only at the dark phase, compared with ND. DIO+TRF significantly reduced vasa recta-associated CD3^+^ cells similar to ND (Figure 2C). The number of cortical tubulointerstitial CD3^+^ cells was increased with DIO, only at the dark phase, and reduced in DIO+TRF. No glomerular-associated CD3^+^ cells were observed at either time point (Figure 2, D and E).

We used the flow cytometry gating scheme shown in Supplemental Figure 2 to assess immune cells — specifically, T cells. We observed higher total immune cells (CD45^+^) at the dark phase with DIO compared with the dark phase with ND (Supplemental Figure 3A). Kidney T cells (TCR-β^+^) were higher in the dark phase of DIO mice compared with the light phase of DIO mice or the dark phase in ND mice (Figure 2F). DIO+TRF significantly decreased T cells compared with DIO (Figure 2F).

We assessed markers of T cell activation/memory (CD44), proliferation (Ki-67), and cytotoxicity (granzyme B and perforin), as well as cytokine production in CD8^+^ T cells by flow cytometry immunophenotyping. At dark phase only, DIO mice had significantly higher CD8^+^ T cells compared with ND, which was significantly reduced in the DIO+TRF group (Figure 2G). CD44^+^CD8^+^ T cells or Ki-67^+^CD8^+^ T cells were increased with DIO in the dark phase compared with ND (Figure 2, H and I). The DIO+TRF group showed significantly reduced CD44^+^CD8^+^ T cells, but not Ki-67^+^CD8^+^ T cells (Figure 2, H and I). We also observed that DIO+TRF significantly reduced TNF-α^+^CD8^+^ T cells at dark phase compared with DIO (Figure 2J) with no effect on IFN-γ^+^CD8^+^ T cells (Figure 2K). Granzyme B^+^CD8^+^ T cells or perforin^+^CD8^+^ T cells were significantly increased with DIO, while DIO+TRF reduced these cells at ZT13–15 (Figure 2, L and M). These data show that TRF intervention in the DIO mice had a time-of-day specific effect on activated, cytotoxic, and proinflammatory CD8^+^ T cells.

We found that, at the dark phase, CD4^+^ T cells were increased with DIO compared with ND but without an effect of TRF compared with DIO (Supplemental Figure 3B). We also assessed markers of T cell activation (CD44) and proliferation (Ki-67) as well as cytokine production and specific subsets of CD4^+^ T cells. There were no significant changes in CD44^+^ or Ki-67^+^ CD4^+^ T cells at either time of day (Supplemental Figure 3, C and D). Th17 and Th1 cells are known to be proinflammatory, whereas Tregs have an antiinflammatory phenotype. At both light and dark phases, there are no significant changes in Th17, Treg, or Th1 cells (Supplemental Figure 3, E–G). These data indicate that TRF intervention in DIO mice does not have any significant effect on kidney CD4^+^ T cells.

We observed no significant changes of circulating CD45^+^ cells, total T cells, CD8^+^ T cells, or CD4^+^ T cells at either time of day or between groups (Supplemental Figure 3, H–K). Immunophenotyping analysis of the small intestine revealed a significant time-of-day difference with higher numbers of CD45^+^ cells, total T cells, CD8^+^ T cells, and CD4^+^ T cells during the dark phase compared with the light phase independent of diet. No significant changes are observed in the small intestine with diet alone (Supplemental Figure 3, L–O).

The Peyer’s patches are lymph node–like structures embedded in the wall of the small intestine and are a key site of T cell priming. There were no significant changes in the frequency of TCR-β^+^ T cells and CD8^+^ T cells between ND, DIO, and DIO+TRF at ZT13–15 (Supplemental Figure 4, A and B). DIO significantly increased the frequency of CD44^+^CD8^+^ T cells compared with ND. DIO+TRF had significantly reduced CD44^+^CD8^+^ T cells in the Peyer’s patches at dark phase compared with DIO group (Supplemental Figure 4C). DIO+TRF significantly reduced granzyme B^+^CD8^+^ T cells with no effect of DIO or DIO+TRF on perforin^+^CD8^+^ T cells in the Peyer’s patches at dark phase (Supplemental Figure 4, D and E). We also assessed T cell responses at dark phase in the mesenteric adipose tissue (MAT), which is the AT surrounding the small intestine and colon. DIO+TRF significantly increased the frequency of total T cells in the MAT compared with DIO (Supplemental Figure 4F). There was no effect of DIO or DIO+TRF on total frequency of CD8^+^ T cells or CD44^+^CD8^+^ T cells in the MAT (Supplemental Figure 4, G and H). DIO significantly increased the percentage of granzyme B^+^CD8^+^ T cells in the MAT compared with ND, while TRF significantly reduced this population compared with DIO (Supplemental Figure 4I). There was no effect of DIO or DIO+TRF on perforin^+^CD8^+^ T cells in the MAT (Supplemental Figure 4J). Lastly, perirenal adipose tissue (PRAT) showed no change between the 3 groups for TCR-β frequency when assessed at dark phase (Supplemental Figure 4K). CD8^+^ T cell frequency was upregulated in both DIO and DIO+TRF compared with ND (Supplemental Figure 4L), but we did not find a change in CD44^+^CD8^+^ T cells (Supplemental Figure 4M). DIO+TRF had significantly reduced granzyme B^+^CD8^+^ T cells with no change in perforin^+^CD8^+^ T cells compared with the DIO group (Supplemental Figure 4, N and O). These data indicate that granzyme B^+^CD8^+^ T cells are responsive to TRF in the DIO model.

### TRF reduces CD8^+^ T cell infiltration into the kidney from the small intestine and associated tissues.

We used photoconvertible KikGR mice to assess cell migration from small intestine and associated tissues (Peyer’s patches and MAT) to the kidney. KikGR mice were placed on ND, DIO, and TRF protocols beginning at 8–10 weeks of age (Figure 3A). At week 20, the small intestine and associated tissues were photoconverted at ZT1–3. After 2.5 days, we harvested tissues at ZT13–15 for analysis of KikRed^+^ cells in the kidney (Figure 3A). We validated our model by collecting tissues immediately after photoconversion to ensure that only the small intestine, Peyer’s patches, and MAT had a positive red signal (Supplemental Figure 5). We found that the frequency of KikRed^+^CD45^+^ cells in the kidney were significantly increased in the DIO group compared with ND, with no effect of TRF (Figure 3, B and C). The frequency of KikRed^+^TCR-β^+^ T cells in the kidney was increased in DIO compared with ND, which was significantly reduced in the DIO+TRF group (Figure 3, D and E). DIO significantly increased KikRed^+^CD8^+^ T cells in the kidney compared with ND, while TRF significantly decreased small intestine–, Peyer’s patch–, and MAT–derived CD8^+^ T cells (Figure 3, F and G). There was no effect of DIO and DIO+TRF on migration of CD4^+^ T cells from the small intestine, Peyer’s patches, and MAT to the kidney (Figure 3, H and I). We analyzed circulating immune cells in this model and found no significant change in KikRed^+^CD45^+^, TCR-β^+^, CD8^+^ T cells, or CD4^+^ T cells (Supplemental Figure 6, A–D). Taken together, these results indicate that CD8^+^ T cells, but not CD4^+^ T cells, are activated to migrate from the small intestine (or associated tissues) to the kidney in DIO and are responsive to TRF.

### Depletion of CD8^+^ T cells in DIO reduces interstitial fibrosis and damage in the kidney.

We reasoned that CD8^+^ T cells were profibrotic in the context of DIO. Mice received 2 weeks of α-CD8 or control α-IgG after 18 weeks of ND or DIO (Figure 4A). Both DIO α-IgG– and α-CD8–treated mice had significantly higher body weights than ND α-IgG mice at the end of the 20-week study (Figure 4B). Kidney weights were similar between the 3 groups (Figure 4C). We assessed T cell phenotypes at ZT13–15. DIO α-IgG mice had significantly higher renal CD8^+^ T cells compared with ND α-IgG mice (Figure 4D). α-CD8 treatment was effective in lowering the circulating and kidney CD8^+^ T cells in DIO mice (Figure 4, D and E). Kidney CD4^+^ T cells were similar between the 3 groups (Supplemental Figure 7A).

MRI was employed to assess kidney fibrosis using ADC and the magnetization transfer ratio (MTR) (Supplemental Figure 7B). We observed decreased ADC in the DIO α-IgG group compared with the ND α-IgG group (Figure 4F). ADC was restored with α-CD8–treated DIO mice (Figure 4F). MTR is the ratio between free water movement and collagen, such that an increased MTR indicates increased fibrosis. MTR is analyzed within regions of the kidney. In both the cortex and outer medulla, MTR was significantly increased in the DIO α-IgG mice compared with ND α-IgG mice, and α-CD8 treatment in DIO significantly reduced MTR (Figure 4, G and H). These MRI assessments indicate that CD8^+^ T cells mediate fibrosis in DIO mice.

We confirmed the MRI results with histological measurements of the kidneys in all 3 groups. Using Masson’s trichrome blue staining, we observed a significant increase in medullary interstitial fibrosis in DIO α-IgG mice compared with ND α-IgG mice that was significantly blunted in DIO mice that received α-CD8 treatment (Figure 4, I and J). DIO α-IgG mice had significantly higher collagen 1 and 3 in the medullary interstitial vasa recta region compared with ND α-IgG mice (Figure 4, K and L). DIO α-CD8 significantly reduced vasa recta collagen 1 and 3 compared with control DIO mice (Figure 4, K and L). We also observed a significant increase in cortical tubulointerstitial fibrosis, spell glomerulosclerosis, and tubular vacuoles with DIO α-IgG compared with ND α-IgG (Figure 4, M–O, and Supplemental Figure 7C). DIO α-CD8 also significantly reduced collagen 1 and 3 in the cortex compared to DIO α-IgG (Figure 4, P and Q). DIO α-CD8–treated mice had significantly reduced cortical tubulointerstitial fibrosis but no significant changes in tubular vacuoles or glomerulosclerosis compared with DIO α-IgG (Figure 4, M–O, and Supplemental Figure 7C). Tubular brush border integrity was significantly improved in DIO α-CD8 mice compared with DIO α-IgG mice, although DIO α-CD8 mice still showed worse brush border integrity than ND α-IgG mice (Supplemental Figure 7D). We observed no change between any of our 3 groups in proteinuria, urinary excretion of neutrophil gelatinase-associated lipoprotein (NGAL), kidney injury marker 1 (KIM-1), and albumin (Supplemental Figure 7, E–H). These studies, taken together, indicate that CD8^+^ T cells mediate fibrosis in the kidneys of DIO mice and TRF blocks fibrosis through diminishing CD8^+^ T cells.

### CD8^+^ T cell activation pathways are altered with DIO and TRF.

We isolated CD45^+^ cells from kidneys collected at ZT13–15 for 10X Genomics scRNA-seq to further understand the transcriptional molecular pathways altered with DIO and DIO+TRF. Thirty distinct clusters of major immune cell populations were revealed, with no significant changes in frequency of overall immune cell types between ND, DIO, and TRF groups (Supplemental Figure 8A). We subclustered all T cells to specifically analyze these populations. We found 3 specific CD8^+^ T cell clusters (named CD8T-1, CD8T-2, CD8T-3), 1 CD4^+^ T cell cluster, 2 clusters with equal expression of CD4 and CD8, and 1 cluster of double-negative (DN) expression of CD4 and CD8 (Figure 5A).

Focusing on the CD8^+^ T cell clusters, DIO specifically expanded the CD8T-1 cluster and contracted the CD8T-3 cluster compared with ND and TRF (Figure 5B). There was no change in the frequency of CD8T-2 cells in either DIO or TRF, while TRF expanded the CD8T-3 population compared with ND and DIO (Figure 5B). CD8T-1 cells had significantly higher expression of chemotactic genes, *Ccr5* (C-C motif chemokine receptor type 5) and *Ccl4* (C-C motif chemokine ligand 4). In addition, T cell functional inhibitory markers, specifically *Pdcd1* (programmed cell death 1), *Ctla4* (cytotoxic T-lymphocyte antigen 4), *Tox* (thymocyte selection-associated HMG bOX), *Lag3* (lymphocyte activation gene 3), and *Maf* (MAF bZIP transcription factor) are upregulated, suggesting that CD8^+^ T cell exhaustion pathways are active in this subset (Figure 5B). Upregulation of the *Il7r* gene in the CD8T-2 cluster suggests a homeostatic function of the CD8T-2 cells (Figure 5B). CD8T-3 cells show high expression of many lectin receptors, similar to NK cells, potentially revealing NK-like functions of CD8T-3 cells (Figure 5B). The CD8T-3 cell population also had high expression of *Itga1* (CD49a) suggesting a resident phenotype (Figure 5B).

Feature plots of CD8T-1 genes, *Ccr5*, *Ccl4*, *Pdcd1*, and Tox, reveal an increased number of cells expressing these genes in DIO compared with ND and TRF (Supplemental Figure 8, B–E). Programmed-death 1 (PD-1, encoded by *Pdcd1*), LAG3, TOX, and CTLA4 were 4 markers revealed from the scRNA-seq analysis that we validated through flow cytometry (Figure 5, D–G). The CD8T-1 cluster revealed the highest number of differentially expressed genes (DEGs) between the DIO versus ND and DIO versus TRF comparisons. The DIO versus ND comparison revealed 114 significant DEGs, and the DIO versus TRF comparison revealed 56 DEGs (Supplemental Figure 8F) in the CD8T-1 cluster. When comparing the DEGs between DIO versus ND and DIO versus TRF, 24 genes overlapped as significantly upregulated or downregulated in both comparisons (Figure 5C). These data reveal that the CD8T-1 cluster is influenced by DIO and TRF in terms of total frequency of cells and DEG transcriptional level. Thus, the CD8T-1 subset of cells are of particular interest for future studies.

The CD8T-2 subset cluster did not change by frequency but was influenced by DIO with regard to DEGs identified. The DIO versus ND comparison showed 21 significant DEGs and pathways in DIO, but only 6 DEGs were revealed between DIO versus TRF (Supplemental Figure 8G). Three genes (*Trbc1*, *Pdcd1*, and *Coch*) overlapped as significantly up- or downregulated between DIO versus ND and DIO versus TRF (Figure 5C and Supplemental Figure 8, H–J). Although the CD8T-3 cluster is TRF responsive by frequency, there were no significant DEGs identified in our comparisons of interest. Taken together, these data expose subcluster-specific responses to DIO and TRF, revealing multiple mechanisms at play.

We observed that CD4^+^ T cells were not TRF responsive in the kidney; however, there was an overall increase in renal CD4^+^ T cell numbers with DIO compared with ND. Therefore, we investigated further transcriptional changes in CD4^+^ T cells with DIO and TRF. There were 35 significant DEGs between DIO versus ND (Supplemental Figure 9A). Interestingly, *Foxp3* (Treg transcription factor) and other Treg-associated genes were significantly upregulated in DIO compared with ND, which was also associated with increased proexhaustion inhibitory markers (Supplemental Figure 9B). These data suggest a potential dysfunction in Treg activity in the DIO kidney. Furthermore, we found that TRF downregulates 9 genes that are linked to T cell dysfunction (Supplemental Figure 9, C and D).

### Representative images and spatial bioinformatic analysis of multiplex immunofluorescence imaging.

We used Lunaphore COMET mulitplex immunofluorescence imaging to spatially analyze T cells, endothelial cells, and collagen-1 in the kidney. We first used Visiopharm analysis tools to create regions of interest (ROIs) for the medulla (NKCC2) and cortex (NCC) (Figure 6A). We show (Supplemental Figure 10, A and B), at 80× magnification, how T cells were defined by double-positive CD45 (cyan) and CD3 (magenta) staining, shows as a white cell in the images. Representative images at 80× magnification are also shown for collagen-1 (red), CD31 (green), CD8a (cyan), and PD-1 (pink) in ND, DIO, and DIO+TRF (Figure 6,B and F). Cells that are double positive for CD8a and PD-1 are white from overlap of cyan and pink. We found that the cell counts of total endothelial cells, total CD8^+^ T cells, and total PD-1^+^CD8^+^ T cells within the medulla and cortex were similar between the ND, DIO, and DIO+TRF groups (Figure 6, C–E and G–I).

We segmented the medulla and cortex into collagen-rich versus collagen-poor areas to identify the numbers of PD-1^+^CD8^+^ T cells and total CD8^+^ T cells. Total CD8^+^ T cells and PD-1^+^CD8^+^ T cells are significantly higher in DIO medullary collagen-rich, while DIO+TRF significantly reduced the number of total and PD-1^+^CD8^+^ T cells (Figure 7A). These cell numbers are all similar in the medullary collagen-poor areas (Figure 7B). No significant changes in total CD8^+^ T cells or PD-1^+^CD8^+^ T cells in the cortical collagen-rich or collagen-poor areas were observed; however, there was a trend of increased PD-1^+^CD8^+^ T cells in DIO within the collagen-poor area that was blunted with DIO+TRF (Figure 7, D and E). Areas within the cortex and medulla differed between each slide based on the depth of the sections. Therefore, we did not observe any differences in the ND, DIO, and DIO+TRF groups in total CD8^+^ T cell numbers in either the medulla or cortex, which does not reflect our flow cytometry data. However, when cell numbers were adjusted for the area in the collagen-rich and collagen-poor regions of the kidney medulla and cortex, we found a significant increase in PD-1^+^CD8^+^ T cells in DIO that is reduced with DIO+TRF in the kidney medulla but not cortex (Figure 7, A, B, D, and E)

To determine the spatial relationship of PD-1^+^CD8^+^ T cells with CD31^+^ endothelial cells, G-function analysis was performed. The G-function or radius quantifies the cumulative infiltration probability between 2 cell types across a range of radii (29), a higher value correlates with an increase in infiltration probability. In the medulla, the G-function analysis revealed a significant relationship between diet and radii with the endothelial cells as the host and PD-1^+^CD8^+^ T cells as the target (Figure 7C). In the cortex, the G-function analysis did not reveal a significant infiltration probability of the target PD-1^+^CD8^+^ T cells and the host CD31^+^ endothelial cells (Figure 7F). These data indicate that DIO leads to significantly increasing numbers of PD-1^+^CD8^+^ T cells infiltrating near endothelial cells as well as within the fibrotic region of the medulla, but not the cortex, that is blocked by TRF. Collectively, these spatial bioinformatic analyses provide mechanistic insight on the distinct localization differences that were identified for PD-1^+^CD8^+^ T cells within the kidney as well as within fibrotic regions of the diet groups.

## Discussion

Mechanistic insight into how TRF improves kidney outcomes in obesity has been lacking. This study conducted in an established DIO mouse model discovered that TRF reverses kidney fibrosis and damage through significantly reducing CD8^+^ T cell infiltration to the kidney as well as showing distinct time of day differences in kidney T cell infiltration and that CD8^+^ T cells migrate from the small intestine (or associated tissues) to the kidney in DIO mice. This study also revealed potential targets for future translational research to reduce the risk of kidney disease in obesity.

The major findings that led to these discoveries are: (a) TRF reverses kidney medullary and cortical interstitial fibrosis in DIO mice assessed by MRI and histological approaches; (b) total T cells and CD8^+^ T cells were significantly increased in DIO mice only in the dark phase while TRF normalized these T cell numbers as evaluated by immunolocalization and flow cytometry; (c) the small intestine and/or associated tissues are a source of kidney infiltrating CD8^+^ T cells in DIO mice while TRF significantly reduces migration of CD8^+^ T cells to the kidney; (d) CD8^+^ T cells mediate kidney interstitial fibrosis and tubular damage in DIO mice; (e) DIO significantly increased kidney CD44^+^CD8^+^, granzyme B^+^CD8^+^, perforin^+^CD8^+^, and TNF-α^+^CD8^+^ T cells while TRF significantly reduced these CD8^+^ T cell phenotypes in the kidney of DIO mice; (f) scRNA-seq of kidney immune cells revealed 3 CD8^+^ T cell clusters with the CD8T-1 cluster expanded in DIO compared with ND and TRF, featuring high expression of exhaustion/activation genes that were validated with flow cytometry, and the CD8T-3 population expanded with TRF in comparison to ND and DIO; and (g) spatial bioinformatic analyses revealed that TRF blocks the increasing numbers of PD-1^+^CD8^+^ T cells infiltrating near endothelial cells as well as within fibrotic regions in the kidney medulla of DIO mice. Overall, this study provides evidence that TRF reverses kidney interstitial fibrosis through reducing CD8^+^ T cell infiltration in obese mice.

### Time of day and T cell localization, phenotyping, and infiltration in the kidney of DIO and TRF mice.

Time-of-day variations in T cells have previously been reported in the blood and lymph nodes (12, 27, 30), but no reports in the kidney or with obesity models are available. We assessed several outcomes in our study in both light and dark phases to define these differences with obesity and following TRF. As expected, light and dark phases showed similar results in structural damage in all groups of mice. Interestingly, T cell responses in the kidney revealed a time-of-day difference with obesity and TRF for localization, phenotyping, and infiltration.

Little is known about T cells in the kidneys of obese mice. However, AT in obese mice has been extensively studied with T cell phenotyping and infiltration. T cells in AT have a proinflammatory phenotype rich with CD8^+^ T cells. CD8^+^ T cells are responsible for recruitment of macrophages to AT, which mediates the proinflammatory state (14). Other studies have shown that AT specific T cells have increased dysfunction in obesity (31, 32). It is unclear how these phenotypes translate to the kidney in DIO. Lee et al. reported that 8 weeks of TRF significantly decreased CD8^+^ T cells, but not CD4^+^ T cells, in AT during DIO (33). However, this study only reported results assumed as during the light phase, but the time was not reported. In an arthritis model, TRF intervention restored diurnal variations of cytokine production in AT as well as adhesion molecule expression and monocyte infiltration in the lung (34). While this study is not an obesity model, it provides context for the rhythmicity of the immune response and TRF.

We found that TRF reduces kidney CD44^+^CD8^+^, granzyme B^+^CD8^+^, perforin^+^CD8^+^, and TNF-α^+^CD8^+^ T cells in the dark phase of DIO mice when compared with DIO ad libitum mice. These data indicate that TRF reduces a population of proinflammatory and cytotoxic CD8^+^ T cells with DIO. Memory CD8^+^ T cells were specifically implicated as profibrotic in the heart and liver (35, 36). The cytotoxicity of CD8^+^ T cells is often promoted by the granzyme/perforin pathway, especially in tumor-infiltrating CD8^+^ T cells. We observed that TRF reduces granzyme B^+^CD8^+^ T cells in the Peyer’s patches, PRAT, and MAT in the dark phase compared with DIO ad libitum mice, indicating that granzyme B abundance is responsive to timing of food intake. Previous work has found that granzyme B is expressed in a diurnal rhythm in the spleens of rats fed a ND (37). Mice kept in constant darkness for 2 days maintained a rhythm of granzyme B abundance in NK cells with a peak at ZT8 (38). These data, taken together, suggest that timing of food intake may be a zeitgeber for granzyme B abundance in T cells.

Immunophenotyping results show a distinct time-of-day difference for both CD8^+^ and CD4^+^ T cells in kidneys from DIO mice. Thus, we reasoned that obesity activates T cell migration and infiltration into the kidney in the dark phase. Endothelial adhesion molecules, vascular cell adhesion molecule 1 (VCAM1) and intracellular cell adhesion molecule 1 (ICAM1), are known regulators of leukocyte migration and infiltration. VCAM1 and ICAM1 are upregulated in AT of humans (39–41) and mice (42, 43) with obesity. VCAM1 and ICAM1 are also both expressed in a diurnal rhythm in the bone marrow, spleen, liver, and lung (27). In addition, homing of leukocytes to the liver, lung, and spleen is time-of-day dependent (27). However, very little is known about whether DIO or TRF affects the abundance and expression of endothelial adhesion molecules in the kidney and whether there is a diurnal rhythm. Future follow-up studies are planned to elucidate these outcomes.

To determine a potential source of the kidney infiltrating CD8^+^ T cells in DIO, we reasoned that the small intestine would be a key source influenced by diets. Our lab previously showed that a high-salt diet promotes mobilization of T cells from the small intestine or associated tissues (19). We now find a significant increase in kidney CD8^+^ T cell infiltration from the small intestine or associated tissues with DIO and blocked with TRF. DIO is well known to dysregulate the diversity and rhythmicity of the gut microbiome (44), while TRF can restore gut microbiome homeostasis (45–47). The gut microbiome may affect immune cell activation and migration from the small intestine (48). New studies are needed to identify how DIO is promoting the increased CD8^+^ T cell migration on the gut-kidney axis, whether endothelial-derived factors, T cell migration markers, and/or the gut microbiome mediate the migration, and how TRF blocks these pathways.

We propose that there is a time-of-day–dependent CD8^+^ T cell egress out of the kidney during the light phase. Sphingosine-1-phosphate receptor 1 (S1PR1) signaling is critical for egress from tissues (49) being previously identified as a critical marker for kidney T cell infiltration in autoimmunity (18). S1PR1 is expressed on a diurnal rhythm, with highest expression in the light phase (12), which may mediate T cell egress. Further studies are needed to identify the time-of-day–dependent kidney T cell migration dynamics in DIO and TRF.

### CD8^+^ T cells mediate kidney fibrosis and damage in DIO.

We discovered that systemic depletion of CD8^+^ T cells in a DIO model reduced kidney fibrosis and tubular damage. The role of CD8^+^ T cells in renal fibrosis is likely context dependent, as there are several conflicting studies using different models (26, 50–53). Previous investigations showed that CD11c^+^CD8^+^ T cells are protective against renal fibrosis in UUO (52). Depletion of CD8^+^ T cells in UUO promoted renal fibrosis (53). The UUO model produces rapid and severe kidney fibrosis, while the DIO model of kidney fibrosis seen in our study is relatively moderate. In models of fibrosis lacking a dietary component may reveal that HFD reprograms renal CD8^+^ T cells to a profibrotic phenotype that is not observed in UUO or other fibrosis models.

Despite several studies suggesting a beneficial role of CD8^+^ T cells in the kidney, some studies suggest pathogenic programming of CD8^+^ T cells in kidney-related disease. Benson et al. demonstrated a proinflammatory role for CD8^+^ T cells directly interacting with the distal convoluted tubule through the programmed death ligand 1 (PDL1) protein critical in the promotion of salt-sensitive hypertension (6). Trott et al. revealed that CD8^+^ T cells mediate the hypertensive response and vascular remodeling in the angiotensin II-induced hypertension model (54). These studies provide a rationale to investigate if CD8^+^ T cells promote tubular or vascular dysfunction in DIO.

Recent studies suggest that fibroblast activation may occur through granzyme B signaling (55), highlighting a potential CD8-dependent target. Granzyme B^+^CD8^+^ T cells mediate cardiac fibrosis in the context of an angiotensin II model of hypertension (56) and after myocardial infarction (57). Granzyme B deficiency improved endothelial barrier function (56). Granzyme B may mediate endothelial dysfunction allowing the infiltration of profibrotic CD8^+^ T cells into the kidney. Granzyme B is necessary for CD8^+^ T cell migration to sites of inflammation (58), further suggesting that granzyme B may mediate both the promigratory and profibrotic phenotype in DIO. It is interesting that T cell granzyme B abundance was reduced by TRF in the majority of the tissues we analyzed. Thus, we propose that granzyme B upregulation mediates the profibrotic phenotype of CD8^+^ T cells in DIO and TRF mitigates this phenotype.

### scRNA-seq identification of markers for CD8^+^ T cell responses in DIO and TRF.

We utilized scRNA-seq analysis to determine pathways in response to DIO and TRF in kidney immune cells. Analysis of the immune cells in total revealed no changes by cluster frequency. Specific T cell subclustering revealed DIO and TRF responses by cluster frequency — specifically CD8T-1, CD8T-2, and CD8T-3. CD8T-1 cluster was expanded by DIO, contracted by TRF, and also showed the highest number of DEGs. CD8T-2 cluster did not have changes in cell frequency between the 3 groups, but DEGs were identified. CD8T-3 cluster was expanded by TRF with no transcriptional changes between the experimental groups. These responses by the CD8^+^ T cell subclusters provide mechanistic insight in how CD8^+^ T cells mediate interstitial fibrosis and how TRF intervention blocks fibrosis.

The CD8T-1 cluster was defined by upregulation of genes linked to CD8^+^ T cell migration and exhaustion (59). The CD8T-1 cluster was expanded by DIO and contracted by TRF to the same level as ND while also showing significant transcriptional changes. We focused on the 24 genes that overlapped as significant between DIO versus ND and DIO versus TRF. Interestingly, we found increased expression of CCR5 (CCL5 and CCL4 receptor) and C-C chemokine ligand 4 (CCL4) in the CD8T-1 subcluster of kidney T cells in DIO compared with ND and DIO+TRF. In DIO, T cells infiltrate the AT in a CCL5-dependent manner (13). Furthermore, increased CCL5/CCR5 in AT of humans and mice is associated with obesity and T cell inflammation (13, 60, 61). Thus, the CCR5 pathway is a potential target for obesity-related kidney disease.

*Coch* was upregulated in DIO in both CD8T-1 and CD8T-2 clusters. *Coch* has been extensively studied as a mediator of hearing loss and autoimmune disease of the ear (62, 63) as a component of the extracellular matrix (ECM) in the inner ear (64). It is reasonable to speculate that *Coch* may be a component of the ECM in other tissues as well. The CD8T-3 cluster has a specific expansion in response to TRF despite the relatively low frequency. The CD8T-3 cluster was defined as the upregulation of many NK-like receptors but also had high expression of *Itga1*, which encodes CD49a. CD49a is a key marker of T cell residency status in the kidney. Other markers, such as CD103 and CD69, will be needed to definitively confirm the resident phenotype. The expansion of CD8T-3 with TRF may indicate that the increased presence of CD11c^+^CD8^+^ T cells mediates reduction in interstitial fibrosis similar to the UUO model. More work is needed to better understand these distinct CD8^+^ T cell phenotypes.

The scRNA-seq findings show increased expression of exhaustion/activation-related markers (*Pd-1*, *Tox*, *Lag3*) on CD8^+^ T cells in DIO mice that are also reduced in response to TRF. We further validated these gene expression results by flow cytometry. Thus, both gene expression and protein abundance of PD-1, LAG3, and TOX are changed with DIO and TRF, which may influence the dysfunction of the CD8^+^ T cells. Exhausted T cells are a dysfunctional state of immune cells that are under chronic antigenic activation (65). Investigations in patients with cancer and models of cancer in preclinical studies show that obesity drives a greater induction of exhausted PD-1^+^ T cells and myeloid cells promoting an immunosuppressive environment (66). With the discovery of the PD-1 pathway and subsequent development of anti-PD-1 therapies or immune checkpoint inhibitors, male patients with obesity who have cancer diagnoses show an improved response rate and survival with these inhibitors (67, 68). PD-1 inhibitors may influence metabolic and immune responses in obesity and kidney disease. PD-1 signaling is also highly relevant to T cell senescence (69). Senescent T cells are dysfunctional and in cell cycle arrest but metabolically active (70). Senescent T cells are known to be important in the progression of kidney disease and hypertension (71, 72). It is possible that the PD-1^+^CD8^+^ T cells are in the senescent stage rather than exhaustion. Further studies will investigate whether the PD-1 pathway in CD8^+^ T cells may be a target in obesity to reduce kidney disease risk.

The spatial bioinformatic analyses provide further mechanistic insight of the PD-1^+^CD8^+^ T cell localizations in response to DIO and TRF within the kidney. The McMahon (73) and Humphries labs (74) utilized spatial transcriptomics in mouse models of acute kidney injury to define injury and repair pathways in specific microenvironments. We utilized spatial bioinformatic analyses of multiplex immunofluorescent localization of the PD-1^+^CD8^+^ T cells. These innovative analyses revealed that PD-1^+^CD8^+^ T cell infiltration probability near the endothelial cells in the kidney medulla of DIO, but not TRF or ND, mice. In addition, the PD-1^+^CD8^+^ T cells are significantly increased in the collagen-rich areas of the kidney medulla of DIO mice. Obesity is known to affect the medulla specifically by mediating alterations in the urine concentrating ability potentially through compression of the thick ascending limb (75, 76). It is unclear how CD8^+^ T cells may affect these medullary-specific mechanisms. These analyses did not reveal similar results in the kidney cortex. A limitation of this analysis in the cortex is that we did not distinguish between the glomerular and interstitial endothelial cells; thus, additional bioinformatic analyses are needed to reveal the specific localizations and cellular interactions occurring in the cortex.

### Perspectives.

This study highlights the importance of time of day, specifically with regard to food intake, as a biological variable and provide mechanistic insight into how TRF is a beneficial intervention in DIO to reduce adverse health outcomes. Panda and colleagues have reported that the window of food intake in human populations is over 16 hours (77, 78). Night-time eating in humans is associated with increased obesity (79), metabolic syndrome (80), and higher risk for cardiovascular and kidney disease (81, 82). Our laboratory previously described that mistimed feeding in mice — that is, when normal, healthy chow is only available during the light phase — caused them to develop significant kidney interstitial fibrosis (83), emphasizing that scheduled eating or time-restricted eating may be a viable lifestyle intervention in obesity to reduce kidney disease.

Potential therapeutic targets were identified for obesity and related kidney diseases in this study. We found that the gut-kidney axis is key in TRF-mediated resolution of the obesity-induced kidney fibrosis and inflammation (84–86). Previous studies report that microbial diversity is lost with DIO and is restored with TRF (47, 87). The gut microbiota is critical for the intestinal immune response (88). Thus, the microbiome and/or microbial-derived metabolites are potential targets for future studies. Knowing that small intestine–derived (or associated tissue–derived) CD8^+^ T cells are mediating adverse outcomes allows for focused research to target gut health in people with obesity as a prevention for the onset of CKD and other obesity-related diseases. Taken together, these studies reveal CD8^+^ T cell responsiveness to DIO+TRF in a time-of-day specific manner that may depend on restoring the migratory rhythm of the gut-kidney signaling axis.

This study focused on the responses of DIO and TRF in male mice. Future research is planned to determine these consequences in female mice. Several labs show that female mice gain weight with high-fat feeding at a slower rate and without the same consequences as males (89, 90). Preliminary findings from our lab have observed that a similar DIO protocol, as is used in this study with males, does not lead to kidney fibrosis in females (91). It is worth noting that, in general, female CD8^+^ T cells exhibit more robust immune responses, higher effector cytokine production, and increased activation compared with males (92–94). In general, males display higher CD8^+^ T cell counts that are susceptible to exhaustion and a tendency to produce greater memory precursor effector cells (92). Since CD8^+^ T cells mediate kidney fibrosis in male DIO mice, CD8^+^ T cells potentially mediate protection against kidney fibrosis in female DIO mice. We propose that these “protective” CD8^+^ T cells in females may be similar to the CD8T-3 cells identified in the scRNA-seq study in the DIO+TRF male mice. Further evaluations are needed to determine whether the CD8^+^ T cell responses in females mediate resistance to kidney fibrosis in DIO.

In conclusion, this mechanistic study shows a dietary and time-of-day influence on CD8^+^ T cells infiltrating to the kidney that mediate fibrosis and damage as well as that TRF reverses kidney fibrosis and damage through diminishing CD8^+^ T cell infiltration. Indeed, this preclinical study reveals that TRF improves DIO-mediated chronic kidney disease risk, providing deeper understanding of the T cell landscape and future targets for translational clinical studies.

## Methods

Supplemental Methods are available online with this article.

### Sex as a biological variable.

Male C57BL6/J (Jackson Laboratory) mice were used in this study. Female C57BL6/J mice have been shown to be resistant to adverse physiological outcomes in DIO (95); thus, we focused this specific project on male mice. It is currently unknown if these findings are relevant to female mice.

### DIO and TRF protocol.

DIO protocols began when mice were 8–10 weeks old. Mice were on 18 weeks of ND (10% fat; 0% sucrose; 3.85 kcal/g; Research Diets, D12450K) or high-fat/sucrose diet (HFD; 45% fat; 20% sucrose; 4.73 kcal/g; Research Diets, D12451) ad libitum. At week 18 of high-fat feeding ad libitum, mice began 2 weeks of TRF or sham intervention. Mice on the TRF protocol did not have HFD available in the light phase from ZT0 (lights on) until ZT12 (lights off). Specifically, mice on the TRF protocol involved research personnel removing food containers to empty the food and replacing empty food containers for 12 hours during the light phase (between ZT0 and ZT12), while the sham protocol involved research personnel removing the food containers and replacing the same containers with replete with food. At ZT12, research personnel again removed the food containers and replaced the same containers replete with food for the dark phase for TRF and sham protocols. Therefore, mice on the TRF intervention only have food available during the dark period, and mice on the sham protocol have food available ad libitum. All TRF and sham mice were exposed to the food containers moving in and out. The mice are denoted as ND, DIO, and DIO+TRF (or TRF). Mouse body weight and food weight were recorded weekly. Mice were group housed with 2–7 mice per cage. Food weight was normalized per mouse and shown per day (g or kcal/d).

### Statistics.

GraphPad Prism 10 or R studio were used for all statistical analyses. Data are all represented as mean ± SEM, and statistical significance is *P* *≤* 0.05. Figure legends specify statistical tests used for each experiment. Two-way ANOVA with a Tukey’s post hoc test was used to assess the effect of time of day and diet groups as well as 1-way ANOVA and 2-tailed *t* test in histology and flow cytometry shown in Figure 1 and Figure 2. One-way ANOVA was used to measure the change between diet groups shown in Figure 3, Figure 4, Figure 5, and Figure 7. Seurat and deSeq were both used for statistical analyses and differential gene expression in R studio shown in Figure 5. Mixed effects analysis with Tukey’s post hoc test was used for G function analysis shown in Figure 7.

### Study approval.

All animal studies were approved by the IACUC at the UAB and were compliant with the *Guide for the Care and Use of Laboratory Animals* (National Academies Press, 2011).

### Data availability.

The scRNA-seq libraries are available at the National Center for Biotechnology Information Gene Expression Omnibus (GEO) under accession no. GSE298793. Specific R code analysis can be found on GitHub. All other data for Figure 5 and Supplemental Figures 8 and 9 are contained within the text and accompanying supplement. All raw data are available in the Supporting Data File.

## Author contributions

CJE and JSP conceived and designed research. CJE, JDE, SCW, XL, JC, PN, MH, APB, and AGS performed experiments. CJE, JDE, PN, KHM, KMK, KH, YHDF, APB, AGS, JLC, and JSP analyzed data. CJE, JDE, DMP, CDM, APB, AGS, JLC, and JSP interpreted results of experiments. SMP and TDR provided training, the KikGR mouse model, and AT flow cytometry with conceptual support and guidance. CDM provided training and expertise in histological analysis and scoring. CJE prepared figures and drafted the manuscript. CJE, KMK, KH, DMP, CDM, AGS, JLC, and JSP edited and revised the manuscript. All authors approved the final submission.

## Conflict of interest

The authors have declared that no conflict of interest exists.

## Funding support

This work is the result of NIH funding, in whole or in part, and is subject to the NIH Public Access Policy. Through acceptance of this federal funding, the NIH has been given a right to make the work publicly available in PubMed Central.

- Voucher award from the UAB Flow Cytometry Core (TDR, DMP, JSP).
- The UAB AMC21 Multi-Investigator Grant (JSP and DMP).
- Diabetes Research Connection (CDM.)
- Hirshberg Foundation Seed Award (JLC).
- Voucher award from the O’Neal Comprehensive Cancer Center (JLC).
- Startup funds from the Department of Medicine/Hematology & Oncology and O’Neal Comprehensive Cancer Center (JLC).
- NIH TL1TR003106 (CJE).
- NIH T32GM8111 (CJE).
- NIH T32 HL007457 (CJE.)
- NIH F31HL167626 (CJE).
- NIH R25DK115353 (JSP and JDE).
- NIH K99HL165091 (KMK).
- NIH R01DK126664 (KH.)
- NIH K01HL145324 (CDM).
- NIH U24DK132740 (CDM).
- NIH P01HL158500 (KH, JSP, DMP).
- NIH P30CA013148 (AGS).
- NIH S10OD028498 (AGS).
- NIH R01CA279143 (AGS).
- NIH R01DK134562 (JSP and DMP).

## Supplementary Material

Supplemental data

Supporting data values

## Figures and Tables

**Figure 1:**
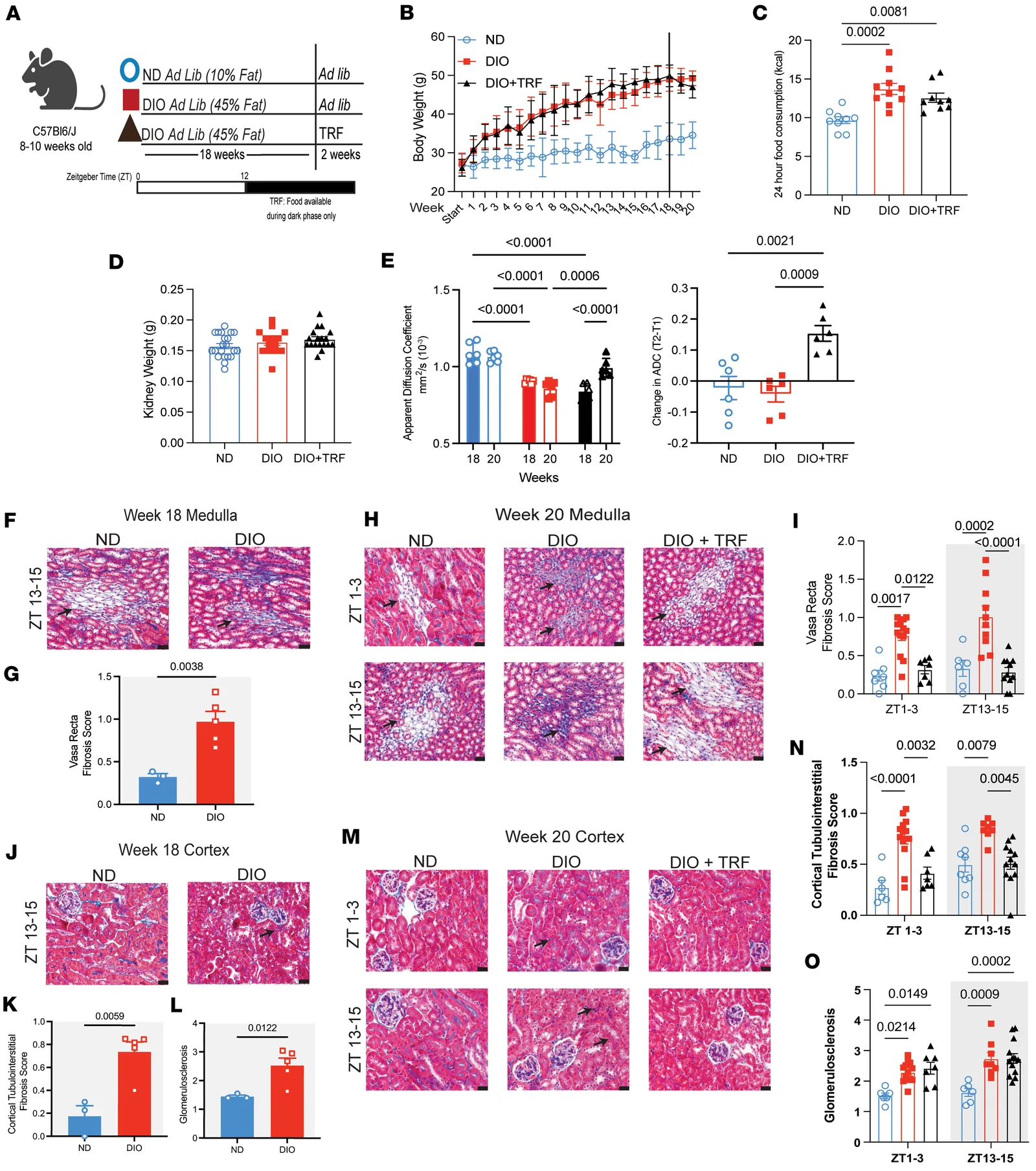
TRF reverses renal fibrosis and tubular damage in DIO mice. (**A**) Experimental design of dietary protocol with 18 weeks of ad libitum feeding before 2 weeks of ad libitum feeding or TRF. (**B**) Longitudinal weight gain, measured weekly, from start of normal or high-fat diet until end of dietary protocol. (**C**) Twenty-four–hour kilocalorie (kcal) intake for ND, DIO, and DIO+TRF groups during 2-week intervention period. (**D**) Kidney weight from all 3 experimental groups at the end of the 20-week protocol. (**E**) ADC shown for ND, DIO, and DIO+TRF at 18 weeks and 20 weeks of diet and change in ADC from 18 weeks to 20 weeks for ND, DIO, and DIO+TRF. (**F**) Representative 40× images of the kidney outer medulla (black arrow pointing to vasa recta bed). (**G**) Histological fibrosis scores for vasa recta fibrosis. (**H**) Representative 40× images of the kidney outer medulla (black arrows pointing to vasa recta bed) stained with Masson’s blue trichrome. (**I**) Histological fibrosis scores for vasa recta associated interstitial fibrosis. (**J**) Representative 40× images of the kidney cortex stained at 18 weeks (black arrows pointing to tubulointerstitial fibrosis). (**K** and **L**) Quantification of cortical tubulointerstitial fibrosis (**K**) and glomerulosclerosis (**L**) at week 18. (**M**) Representative 40× images of the kidney cortex stained at 20 weeks (black arrows pointing to tubulointerstitial fibrosis). (**N** and **O**) Quantification of cortical tubulointerstital fibrosis (**N**) and glomerulosclerosis (**O**) at week 20. Two-way ANOVA was used to compare time of day and dietary groups with Tukey’s post hoc test (**E**, **I**, **N**, and **O**); 1-way ANOVA in **C**–**E**; and 2-tailed *t* test in **G**, **K**, and **L**. Gray background is used for ZT13–15 (dark phase); white background is used for ZT1–3 (light phase). Scale bars are 20 µm.

**Figure 2:**
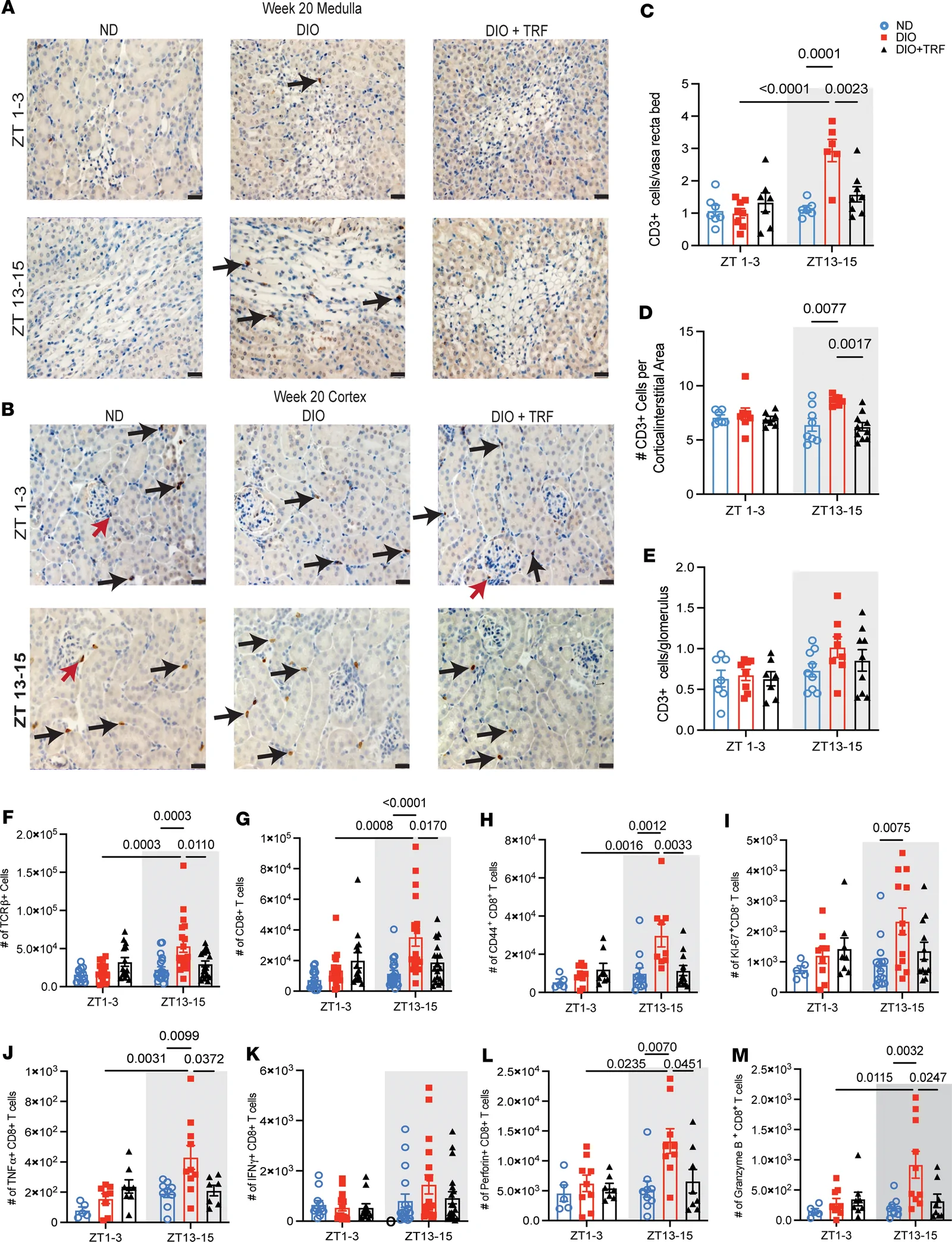
TRF reduces dark phase renal CD8^+^ T cells in DIO mice. (**A** and **B**) Representative 40× images of the kidney outer medulla (**A**, black arrow indicates CD3^+^ staining) and cortex (**B**, black arrow indicates interstitial CD3^+^ staining, red arrow indicates glomerular associated CD3^+^ staining) stained with antibody for CD3. (**C**–**E**) Counts of T cells per vasa recta (**C**), tubular associated T cells per 40× image (**D**), and glomerular associated T cells per glomerulus (**E**). (**F**–**M**) Flow cytometry was performed at ZT1–3 (light phase) and ZT13–15 (dark phase) in all 3 groups to assess TCR-β^+^ T cells (**F**), CD8^+^ T cells (**G**), CD44^+^CD8^+^ T cells (**H**), KI-67^+^CD8^+^ T cells (**I**), TNF-α^+^CD8^+^ T cells (**J**), IFN-γ^+^CD8^+^ T cells (**K**), granzyme B^+^CD8^+^ T cells (**L**), and perforin^+^CD8^+^ T cells (**M**). Two-way ANOVA was used to compare time of day and dietary group with Tukey’s post hoc test (*n* = 3–20, *P < 0.05). Gray background is used for ZT13–15 (dark phase), white background is used for ZT1–3 (light phase). Scale bars are 20 µm.

**Figure 3:**
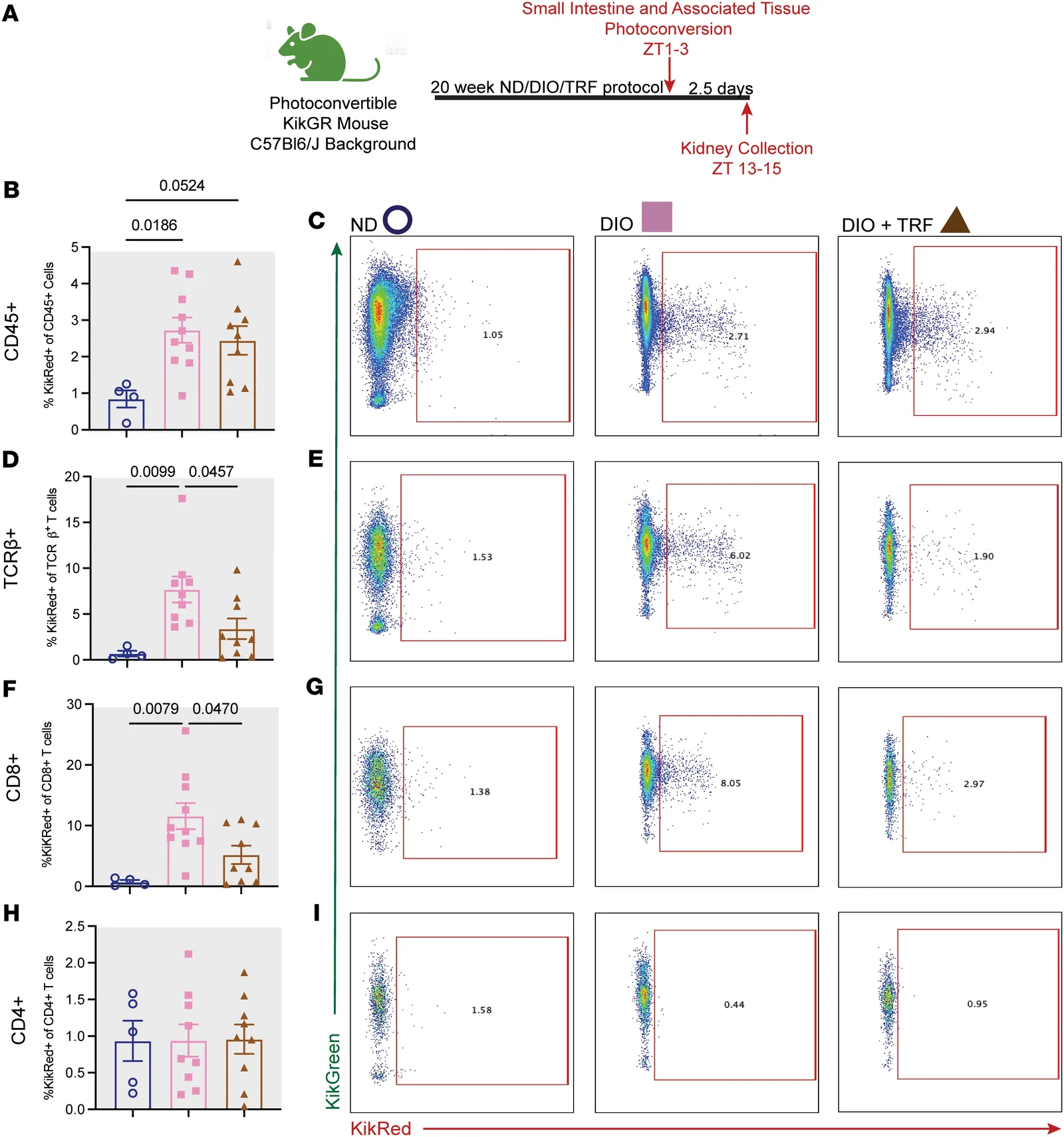
TRF reduces renal CD8^+^ T cell infiltration from the small intestine, Peyer’s patches, and mesenteric adipose tissue in DIO mice. (**A**) Representative scheme of 20-week DIO/TRF with photoconvertible Kikume GreenRed (KikGR). Small intestine and associated tissues (Peyer’s patches and mesenteric adipose) were photoconverted at ZT1–3 on week 20, and 2.5 days later, the kidneys and blood were collected for flow cytometry analysis. (*n* = 4–7.) (**B**–**I**) Flow cytometry data (**B**) and representative flow plots (**C**) of infiltrating CD45^+^ cells, flow cytometry data (**D**) and representative flow plots (**E**) of infiltrating TCR-β^+^ cells, flow cytometry data (**F**) and representative flow plots (**G**) of infiltrating CD8^+^ T cells, and flow cytometry data (**H**) and representative flow plots (**I**) of infiltrating CD4^+^ T cells. One-way ANOVA was used to compared dietary intervention. Gray background is used for ZT13–15 (dark phase).

**Figure 4:**
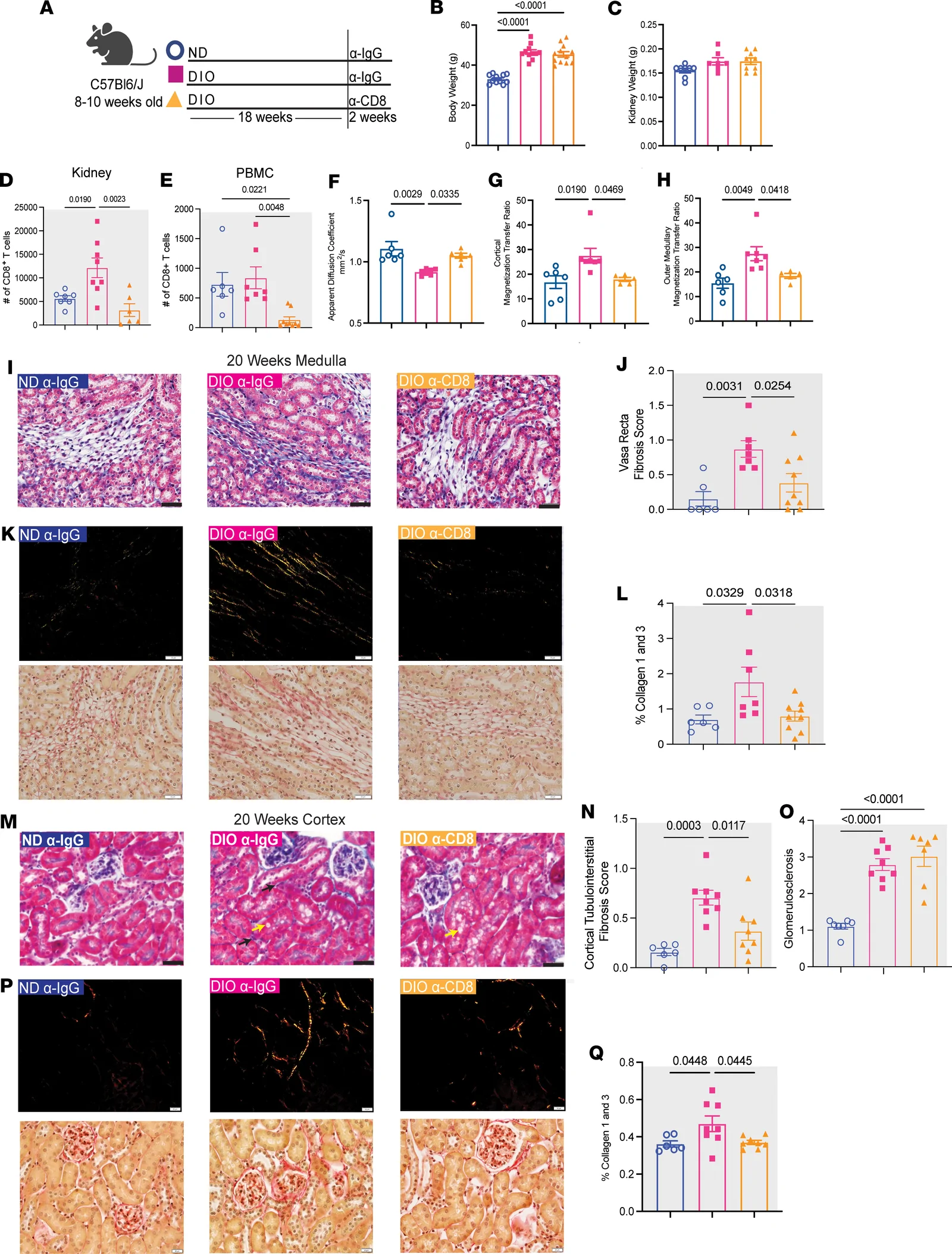
CD8^+^ T cell depletion in DIO mice reduces renal fibrosis and tubular damage. (**A**) Dietary protocol with 18 weeks of ND or HFD feeding with 2 weeks of control anti-IgG or experimental anti-CD8 treatment. (**B** and **C**) Body weights (**B**) and kidney weights (**C**) at conclusion of 20-week protocol. (**D** and **E**) Flow cytometry analysis to confirm effectiveness of anti-CD8 treatment in kidney (**D**) and peripheral blood mononuclear cells (**E**). (**F**) Quantitative ADC measurements for the whole kidney. (**G** and **H**) Quantitative MTR measurements for the kidney cortex (**G**) and outer medulla (**H**). (**I** and **J**) Representative 40X images of the kidney outer medulla with Masson’s trichrome blue staining (**I**) and histological scores of vasa recta interstitial fibrosis at ZT13–15 (**J**). (**K**–**N**) Representative 40X images of the kidney outer medulla with picrosirius red staining (**K**) and quantification of polarized light stain (collagen 1 and 3) (**L**). Representative images of kidney cortex stained with Masson’s blue trichrome with black arrows denoting tubulointerstitial fibrosis and yellow arrows denoting vacuoles (**M**) and histological scoring for cortical tubulointerstitial fibrosis (**N**) and glomerulosclerosis (**O**) in all 3 groups. Representative 40× images of kidney cortex with picrosirius red staining with polarized and nonpolarized light (**P**) and quantification of polarized light stain (collagen 1 and 3) in all 3 groups (**Q**). One-way ANOVA compared dietary intervention (*n* = 7–8). Gray background used for ZT13–15 (dark phase); white background used for ZT2-10 (light phase). Scale bars are 20 µm.

**Figure 5:**
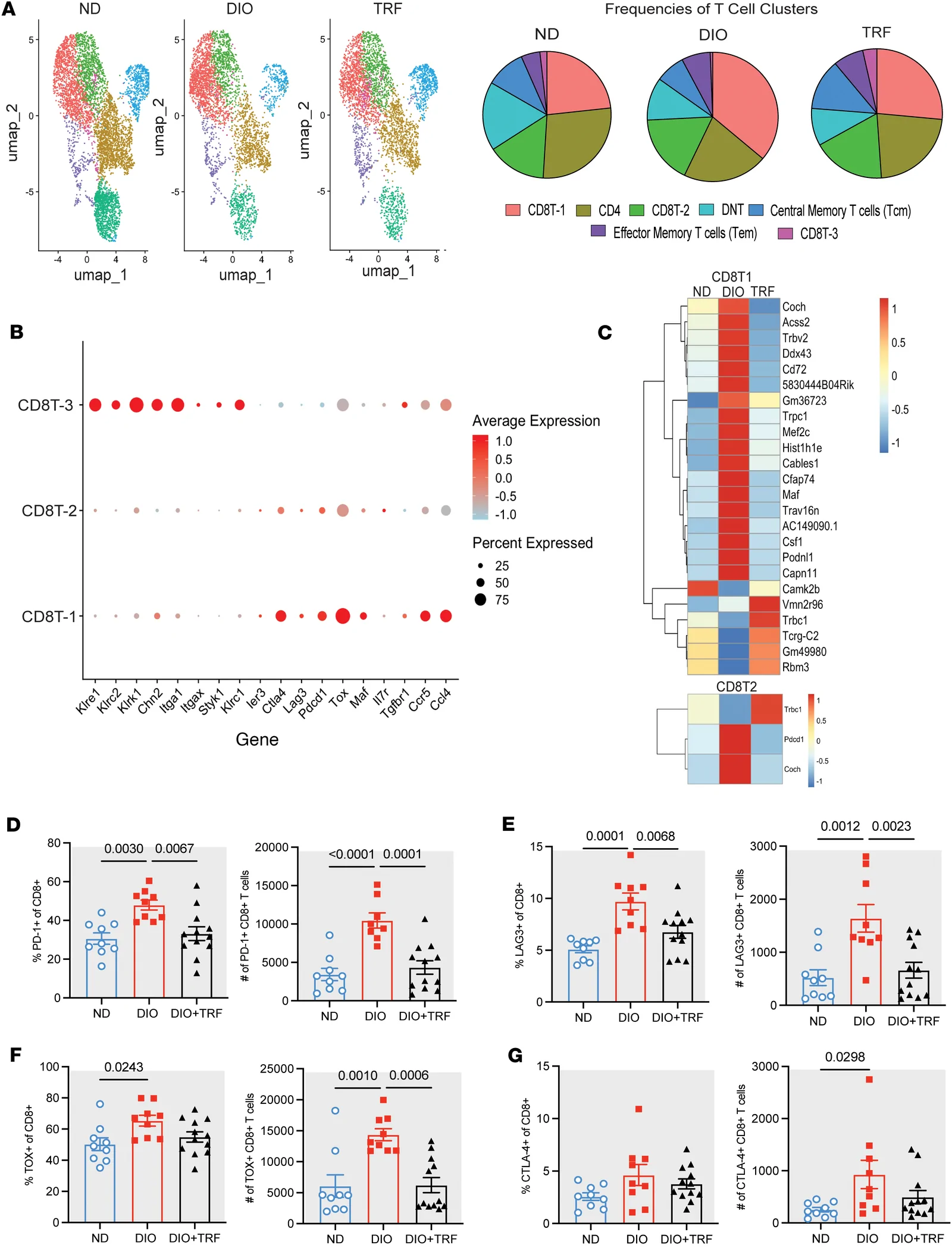
TRF reduces CD8^+^ T cell dysfunction in DIO mice. (**A**) UMAP of T cell cluster subsets from ND, DIO, and DIO+TRF (left) and pie chart showing frequency of each T cell cluster in ND (left), DIO (middle), and DIO+TRF (right). (**B**) Dot plot for DEGs for cluster differentiation for CD8T-1, CD8T-2, and CD8T-3. (**C**) Heatmap of 24 genes that were significantly different between DIO and ND and DIO versus TRF in CD8T-1 subcluster (left) and heatmap of 3 genes that were significantly different between DIO and ND and DIO versus TRF in CD8T-2 subcluster. (**D**–**G**) Flow cytometry analysis for frequency and cell number of PD-1^+^ (**D**), LAG3^+^ (**E**), TOX^+^ (**F**), and CTLA4^+^ (**G**) CD8^+^ T cells. Flow cytometry data were analyzed by 1-way ANOVA (*n* = 9–12). Gray background used for ZT13–15 (dark phase).

**Figure 6:**
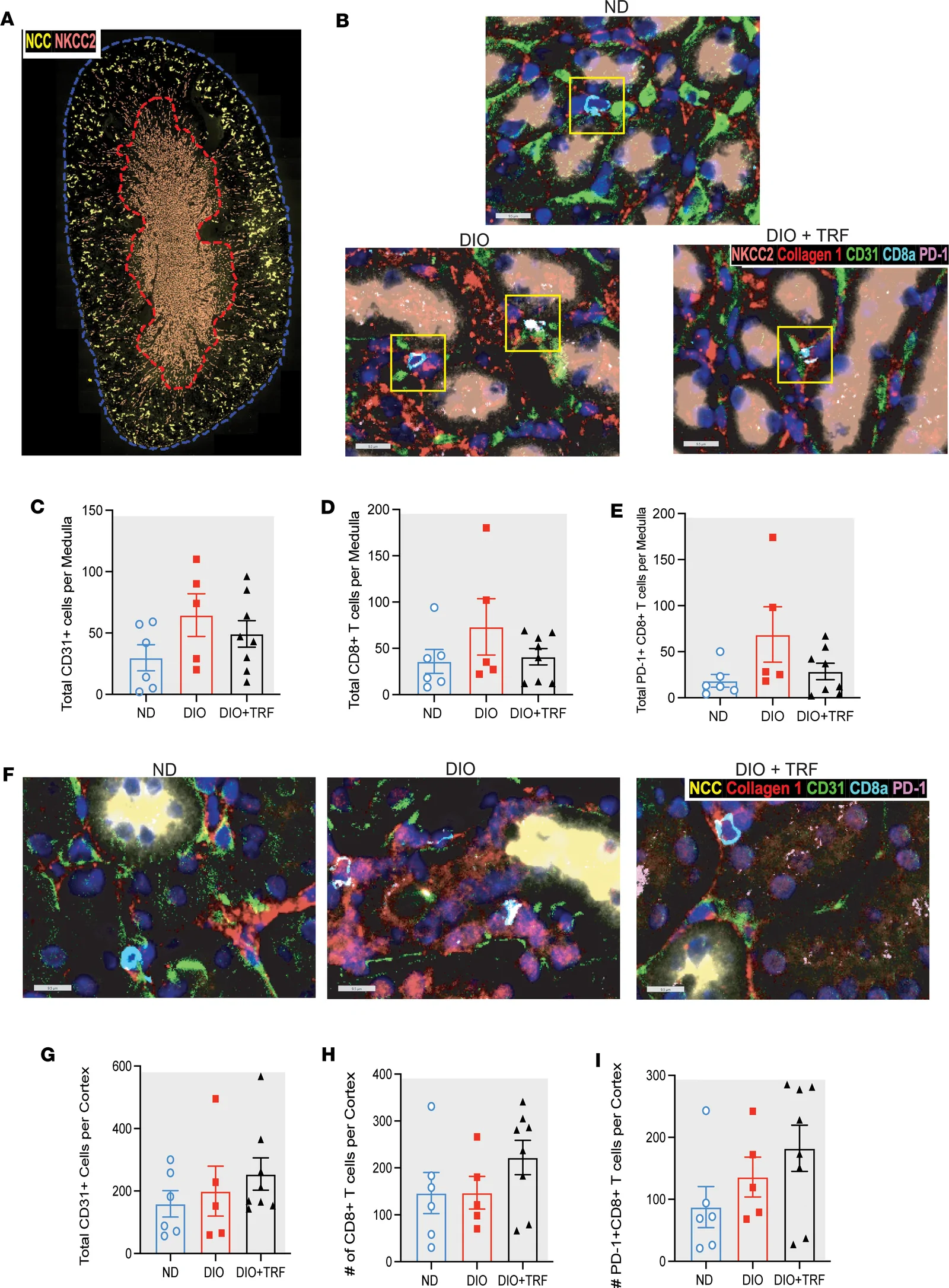
Lunaphore COMET representative images to spatially analyze PD-1^+^CD8^+^ T cells, CD31^+^ cells, and collagen in the medulla and cortex. (**A**) Image of whole kidney with ROI to show cortex via yellow NCC staining (blue dotted line to red dotted line) and medulla via orange NKCC2 staining (inside red dotted line). (**B**) Medulla (NKCC2) representative images (80X) showing collagen-1 (red), CD31^+^ (green), CD8a^+^ (cyan), and PD-1^+^ (pink) in ND, DIO, and DIO+TRF. (**C**–**E**) Total medullary endothelial cells (**C**), CD8^+^ T cells (**D**), and PD-1^+^CD8^+^ T cells (**E**) in ND, DIO, and DIO+TRF. (**F**) Cortex (NCC) representative images (80X) showing collagen-1 (red), CD31^+^ (green), CD8a^+^ (cyan), and PD-1^+^ (pink) in ND, DIO, and DIO+TRF. (**G**–**I**) Total medullary endothelial cells (**G**), CD8^+^ T cells (**H**), and PD-1^+^CD8^+^ T cells (**I**) in ND, DIO, and DIO+TRF. Kidneys were dissected and fixed in the dark phase.

**Figure 7:**
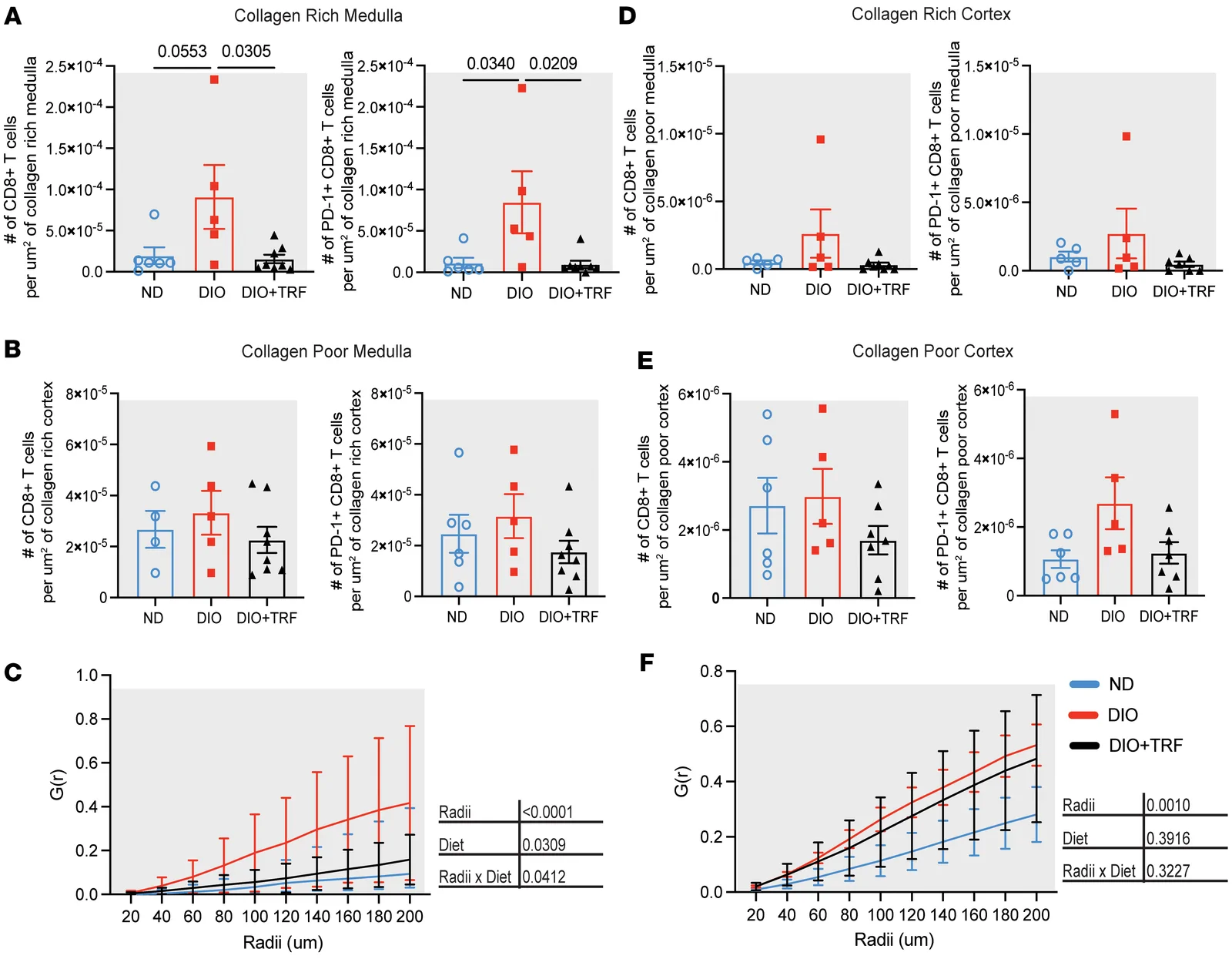
Spatial bioinformatic analysis reveals that TRF significantly reduces PD-1^+^CD8^+^ T cell infiltration probability to CD31^+^ endothelial cells in the medulla only. (**A**) Total CD8^+^ T cells and PD-1^+^CD8^+^ T cells in the collagen-rich area of medulla. (**B**) Total CD8^+^ T cells and PD-1^+^CD8^+^ T cells in the collagen-poor area of medulla. (**C**) G function or G(r) analysis of spatial relation of medullary endothelial cells (host cell) to medullary PD-1^+^CD8^+^ T cells (target cell) in ND, DIO, and DIO+TRF. (**D**) Total CD8^+^ T cells and PD-1^+^CD8^+^ T cells in the collagen-rich area of cortex. (**E**) Total CD8^+^ T cells and PD-1^+^CD8^+^ T cells in the collagen-poor area of cortex. (**F**) G function or G(r) analysis of spatial relation of cortical endothelial cells (host) to cortical PD-1^+^CD8^+^ T cells (target) in ND, DIO, and DIO+TRF. Data analyzed by mixed effects in **C** and **F** (*n* = 5–8). Data analyzed by 1-way ANOVA in **A**, **B**, **D**, and **E**.

## Version 1 08/28/2026

In-Press Preview

## Version 2 10/08/2026

Electronic publication
