# Supplementary material for: Time-restricted feeding reverses kidney fibrosis through reducing CD8+ T cell infiltration in obese mice
Source: JCI Insight. 2026 Aug 20;11(19):e195335. doi: 10.1172/jci.insight.195335 (PMC13726415; doi:10.1172/jci.insight.195335)
Supplement: Supplementary file 1 [file jciinsight-11-195335-s039.pdf]

SUPPLEMENT

TIME-RESTRICTED FEEDING REVERSES KIDNEY FIBROSIS THROUGH REDUCING CD8^{+} T CELL INFILTRATION IN OBESE MICE

Claudia J. Edell[1,2], John D. Erickson[1,2], Xiaofen Liu[1,2], Savannah C. Walker[1,2], Jackson Colson[1,2], Michael Heim[1,2], Pranav Nagila[1,2], Kyle H. Moore[1,2], Keri M. Kemp[1,2], Kelly A. Hyndman[1,2], Selene Mesa-Perez[3], Troy D. Randall[3], Annye Bennett[4], Anna Sorace[4], Yu-Hua Dean Fang[4,5], David M. Pollock[1,2], Carmen De Miguel[1,2], Julienne L. Carstens[6], Jennifer S. Pollock[1,2,7]

[1]CardioRenal Physiology & Medicine, [2]Division of Nephrology Department of Medicine, [3]Division of Clinical Immunology & Rheumatology Department of Medicine, [4]Department of Radiology, [5]Department of Neurology, [6]Department of Hematology and Oncology Heersink School of Medicine, University of Alabama at Birmingham, Birmingham, AL

[7]Corresponding author
Jennifer S. Pollock, PhD
Endowed Professor of Nephrology
McCallum Research Building 515
1918 University Blvd.
University of Alabama at Birmingham
Birmingham, AL 35233
jenniferpollock@uabmc.edu
205-975-7525

Supplemental Methods:

Mice: Specific pathogen free (SPF) C57Bl6/J mice were ordered from Jackson Laboratories at 3 weeks of age (weaning age). Mice arrived at the University of Alabama at Birmingham (UAB) placed in a temperature and humidity-controlled environment in standard cages and were acclimated in the UAB animal facility for 5-7 weeks before protocols were initiated. Mice were provided *ad libitum* standard laboratory chow and *ad libitum* water on a 12:12 light/dark cycle ZT 0 (7am): lights on; ZT12 (7pm): lights off. Mice were monitored by lab and animal facility staff at UAB. Breeding pairs of Kikume Green (KikGreen) mice were kindly provided by Dr. Troy Randall (University of Alabama at Birmingham, Birmingham, AL).

Histological Analysis – Paraffin: Kidneys were collected and immediately fixed in 10% formalin buffer. Tissues were fixed for 24 hours then transferred to 70% ethanol for dehydration. All tissues were sent to UAB Histology Core Facility for paraffin embedding. Tissues were sectioned and stained for fibrosis (Trichrome Blue, Picrosirius Red) and T cell analysis (CD3). Using Odyssey microscope at 40X magnification, 15-20 images were taken per kidney section. Each image was scored using systems described below and scores for each individual image were averaged together for each individual sample. Cortical and outer medullary fibrosis was scored on a scale from 0-2. A score of 0 indicates to no fibrosis present, 1 is intermediate fibrosis, and 2 is high levels of fibrosis. Glomerulosclerosis was scored on a scale from 0-4, with 0 indicating no presence of glomerulosclerosis, 1 indicating about 1 quarter of the glomerulus contains fibrosis, 2 is two quarters, 3 is 3 quarters, and 4 is the whole glomerulus is fibrotic. Brush border integrity is scored on a scale from 1-3, with 3 being indicative of a fully intact brush border,

2 is some disruption and thinning of the brush border, and 1 is a complete loss of the brush border.

Renal immune cell isolation: Kidneys are collected in RPMI-1040 with 10% fetal bovine serum (FBS) and dissociated with Miltenyi gentleMACS dissociater on setting 1. Kidneys were then digested for 45 minutes with collagenase IV (Thermo Fisher, catalog # 17104019), DNAse I (Millipore Sigma, catalog # 10104159001), and Dispase II (Thermo Fisher, catalog # NC9886504) and then dissociated once more on setting 2. Samples were spun at 10g for 1 minute to collect minimize debris when filtering. Samples were filtered through a 70um filter, then a 40um filter. Samples were spun at 400g for 7 minutes. ACK-Lysis buffer was added to each sample for 4 minutes and then spun again at 400g for 7 minutes. Each sample was resuspended in 37% isotonic percoll and spun at 600g for 20 minutes with 0 acceleration or brake. Samples were then resuspended in IMDM with 10% FBS for stimulation if necessary or FACS buffer to move to flow cytometry staining protocols.

KikGreen Photoconversion: The KikGR mouse model is on a C57Bl6/J background and has uniquitous expression of the photoconvertible KikGreen. When exposed to 405nm blue light, peptide bonds break to express the KikRed protein. We put our KikGR mice on our 20-week DIO/TRF protocol. Photoconversion of small intestine protocol was adapted from previously published and validated studies from our lab and others. KikGR male mice were anesthetized with 2-2.5% isoflurane on a warming platform (37°C). Hair removal cream was applied to the abdomen of the mouse for 1-3 minutes. Betadine was then applied to the abdomen to prepare the sterile environment. A small incision was made in the center of the abdomen. The small intestine, mesenteric adipose tissue, and

Peyer's patches (SI+) was withdrawn from the abdomen and gently placed on sterile aluminum foil that covered the whole mouse. A Dymax QX4 405nm wand system with an LED head and collimator was placed 35mm above the small intestine. The front and back of SI+ tissue was exposed to 405nm light at an intensity of 95 mW/cm^2 for 10 minutes (5 minutes per side). SI+ was kept moist with sterile PBS throughout photoconversion period. SI+ was gently tucked back in to abdominal cavity. Muscular and skin layers were closed with sutures. Mice were placed under red heat lamp for about 20 minutes and received PBS dose for hydration. Mice were checked on until sacrifice 2.5 days post-surgery.

Flow Cytometry: If cytokine staining is needed, cells are stimulated for 4 hours with phorbol myristate acetate (PMA) and ionomycin. After one hour, brefeldin A (BFA) is added for the remaining 3 hours. After stimulation, cells are stained with Zombie NIR in PBS to determine live cells for 15 minutes at 4°C. Fc block is added to all cells and incubated for 15 minutes at 4°C before adding surface markers (CD45, TCRβ, CD4, CD8a, CD11b, CD11c, CD44 (Supplemental Table 1)) for 20 minutes at 4°C. If no intracellular staining is needed, cells are fixed with 2% paraformaldehyde (PFA) in FACS buffer. If intracellular staining is needed, FoxP3 Transcription Factor fixability kit (Thermo Fisher/Invitrogen – Catalog #: 00-5523-00) is used overnight to permeabilize and fix the cells. Intracellular cytokines and transcription factors (Il-17A, IFNγ, Granzyme B, Perforin, FoxP3, RORγt, Tbet, TNFα) are added to the cells in 1X permeabilization buffer for 45 minutes at room temperature. Cells are washed and fixed in FACS buffer with 2% PFA. In experiments where KikGR mice are used, KikGreen signal is detected using the 488nm laser (BP 515/20, LP 505), and KikRed signal is detected using the 561nm laser (BP

586/15, LP 570) on the BD FACSymphony A3/A5. KikGreen experiments were acquired on BD FACSymphony and all other flow cyometry experiments were acquired on a Cytek Northern Lights.

CD8 T cell depletion: CD8 T cells were depleted *in vivo* beginning at week 18 of the DIO protocol. Mice are given a 100ug intraperitoneal (IP) injection of anti-CD8 (BioXcell, catalog # BP0117) or anti-IgG (BioXcell, catalog # BP0090) at ZT 12 every 72 hours for 2 weeks.

Renal immune cell isolation: Kidneys are collected in RPMI-1040 with 10% fetal bovine serum (FBS) and dissociated with Miltenyi gentleMACS dissociater on setting 1. Kidneys were then digested for 45 minutes with collagenase IV (Thermo Fisher, catalog # 17104019), DNAse I (Millipore Sigma, catalog # 10104159001), and Dispase II (Thermo Fisher, catalog # NC9886504) and then dissociated once more on setting 2. Samples were spun at 10g for 1 minute to collect minimize debris when filtering. Samples were filtered through a 70um filter, then a 40um filter. Samples were spun at 400g for 7 minutes. ACK-Lysis buffer was added to each sample for 4 minutes and then spun again at 400g for 7 minutes. Each sample was resuspended in 37% isotonic percoll and spun at 600g for 20 minutes with 0 acceleration or brake. Samples were then resuspended in IMDM with 10% FBS for stimulation if necessary or FACS buffer to move to flow cytometry staining protocols.

Single cell RNA sequencing: Kidneys were harvested from mice at ZT13. Kidneys were incubated with collagenase IV, DNAse I, and Dispase II for 15 minutes. Kidneys were then disassociated with Miltenyi gentleMACS dissociate on setting 2. Cells were filtered and red blood cells were lysed before moving to staining. Cells were stained with Zombie NIR

for live/dead analysis and CD45 in brilliant violet (BV) 785. Cells were also incubated with Total-SeqB hashing antibodies per manufacturer's directions. Samples were then FACS sorted for CD45+ cells using the BD S6 6-way sorter. CD45+ cells were sequenced with 10x genomics in collaboration with the UAB Flow Cytometry Core. Data was processed using Cell Ranger. R code used for analysis can be found via GitHub.

Lunaphore COMET Multiplex Immunofluorescence Imaging: Formalin Fixed Parafin Embedded (FFPE) kidneys from ZT13-15 were sectioned and processed for high throughput sequential immunofluorescence (seqIF) staining of 25 targets in collaboration with the UAB Flow Cytometry Core Facility. The stains included in this manuscript include CD45, CD3, CD8, PD-1, CD31, Collagen 1, NKCC2, and NCC. All data was analyzed using the Visiopharm analysis software. Apps were created in Visiopharm to identify tissue regions of interest (ROIs, kidney cortex and kidney medulla), further subset each ROI in to collagen-rich and collagen poor regions, segment cells in each ROI based on NaK-ATPase, and then further phenotyped cells of interest based on specific markers. Spatial clustering of PD-1+ CD8+ T cells and endothelial cells (CD31+) was assessed using the nearest-neighbor distance distribution function G(r), which estimates the cumulative distribution of distances from each point to its nearest neighboring point.

Magnetic Resonance Imaging (MRI): Mice from the DIO/TRF and the DIO/α-CD8 protocol were imaged using 9.4T Bruker small animal preclinical MRI to measure magnetization transfer ratio (MTR) and apparent diffusion coefficient (ADC). Respiratory gating with a respiratory pad was used in combination with our small animal monitoring system that only captures images in-between breaths of the mice. This gating was used for the T2 anatomical scans of the kidneys and the EPI DWI scans. There were 4 B values used for

the DWI scans which were 23.643027 s/mm^2, 166.701866 s/mm^2, 516.701866 s/mm^2, and 816.701866 s/mm^2.

MTR measures the ratio of free water movement and macromolecules, such as collagen. Higher MTR values indicate greater fibrosis and is analyzed per kidney region. ADC is the measurement of water movement throughout the kidney. Lower ADC indicates more fibrosis and is the ADC is based on the whole kidney. The analysis for the ADC and MTR was done volumetrically. ROIs were drawn on the matching T2 anatomical scans of the kidneys, which were 256x256x15 volumes. The ADC maps generated were scaled so the 64x64x15 volume became 256x256x15 so the ROIs drawn can be overlayed on the ADC maps. The MTR had a matching image size originally (256x256), so the ROIs were simply placed onto the MTR maps. The MTR images could only include 5mm of the kidneys, so these were the central slices of each kidney, while the ADC images included the entirety of the mouse's kidneys.

The ROIs were drawn manually on the anatomical images using visible renal anatomical landmarks. The renal cortex was identified as the outer peripheral layer of the kidney, which had a distinct separation between the cortex and outer medulla. The inner medulla was identified with its distinct hyperintense signal, and the outer medulla was defined as the space between the cortex and inner medulla. These ROIs were drawn on both kidneys in all of the mice, and the data was collected volumetrically for each mouse. The ROIs were also drawn to not include the edge of the kidneys to reduce partial-volume artifacts, along with avoiding any major vessels within the kidneys in all of the mice.

Flow Cytometry Antibodies Used:

**Target**	**Clone**	**Company/Catalog #**
CD45	30-F11	Biolegend - 103149
TCRB	H57-597	BD Horizon - 563135
CD11b	M1/70	Biolegend - 101228
CD11c	N418	Thermofisher - 45-0114-82
CD4	GK1.5	Biolegend - 100422
CD8a	53-6.7	Thermofisher - 47-0081-82
Perforin	S16009A	Biolegend - 154306
Granzyme B	GB11	Biolegend - 515406
IFNY	XMG1.2	BD Horizon - 554411
TNFα	MP6-xT22	Biolegend - 506333
CD44	IM7	Biolegend - 103026
CD69	H1.2F3	Biolegend - 104558
CD197 (CCR7)	4B12	Biolegend - 120125
IL-17A	TC11-18H10	BD Horizon - 564170
KI-67	SolA15	Invitrogen - 61-5698-82
CXCR3 (CD183)	CXCR3-173	Biolegend - 126529
FoxP3	FJK-165	Thermofisher - 48-5773-82
RORγt	AFKJ5-9	Thermofisher - 12-6988-82
Zombie NIR	N/A	Biolegend - 432106
TruStain FcX™ (anti-mouse CD16/32)	93	Biolegend - 101320
CD223 (LAG-3)	C9B7W	Biolegend - 125224
CD152 (CTLA-4)	UC10-4B9	ThermoFisher - 17-1522-82

TOX	TXRX10	ThermoFisher - 50-6502-82
CD279 (PD-1)	J43	ThermoFisher - 11-9985-82

Antibodies Used for COMET:

**Target**	**Clone**	**Company/Catalog #**
CD45	Polyclonal	Abcam - ab10558
CD3	CD3-12	Abcam - ab11089
CD8	4SM16	Invitrogen - 14-0195-82
PD-1	D7D5W	Cell Signaling Tech - 84651
CD31	Polyclonal	Abcam - ab28364
Collagen 1	Polyclonal	Southern Biotech - 1310-01
SLC12A3 (NCC)	EPR27106-48	Abcam - ab316191
SLC12A1 (NKCC2)	EPR28176-90	Abcam - ab313640

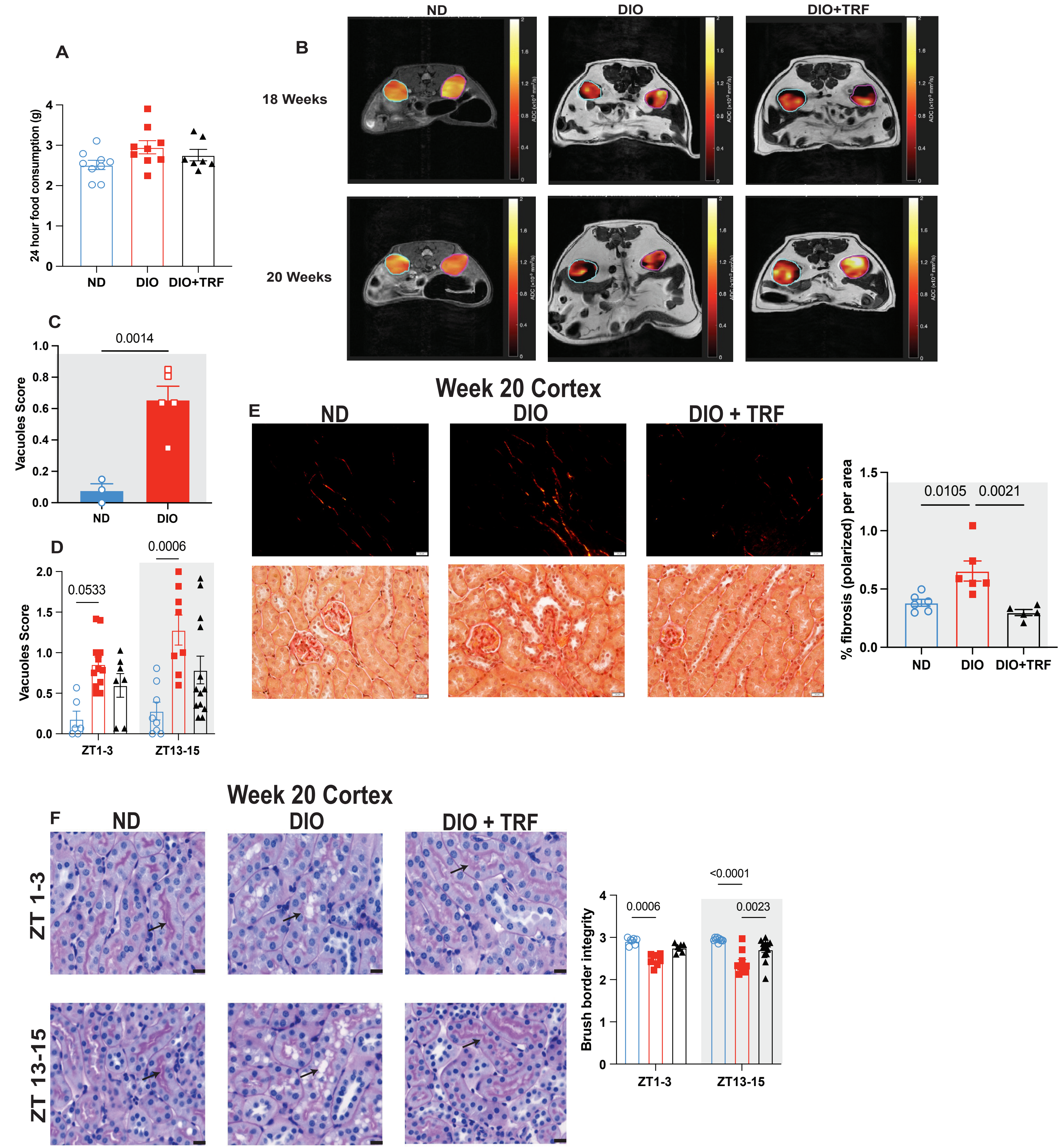

Supplemental Figure 1: Total food consumption between ND, DIO, and DIO+TRF in grams (A). Representative MRI images of ADC measurements at 18 weeks (top) and 20 weeks (bottom) in ND, DIO, and DIO+TRF (B). Quantification of proximal tubular vacuoles in ND and DIO mice at week 18 at ZT13-15 (C) and in ND, DIO, and DIO+TRF mice at ZT1-3 and ZT13-15 (D). Representatve images and quantification of fibrosis with picrosirius red staining in the kidney cortex in ND, DIO, and DIO+TRF mice at ZT13-15 (E). Represenative images and quanification of proximal tubule brush border integrity at ZT1-3 and ZT13-15 (F).

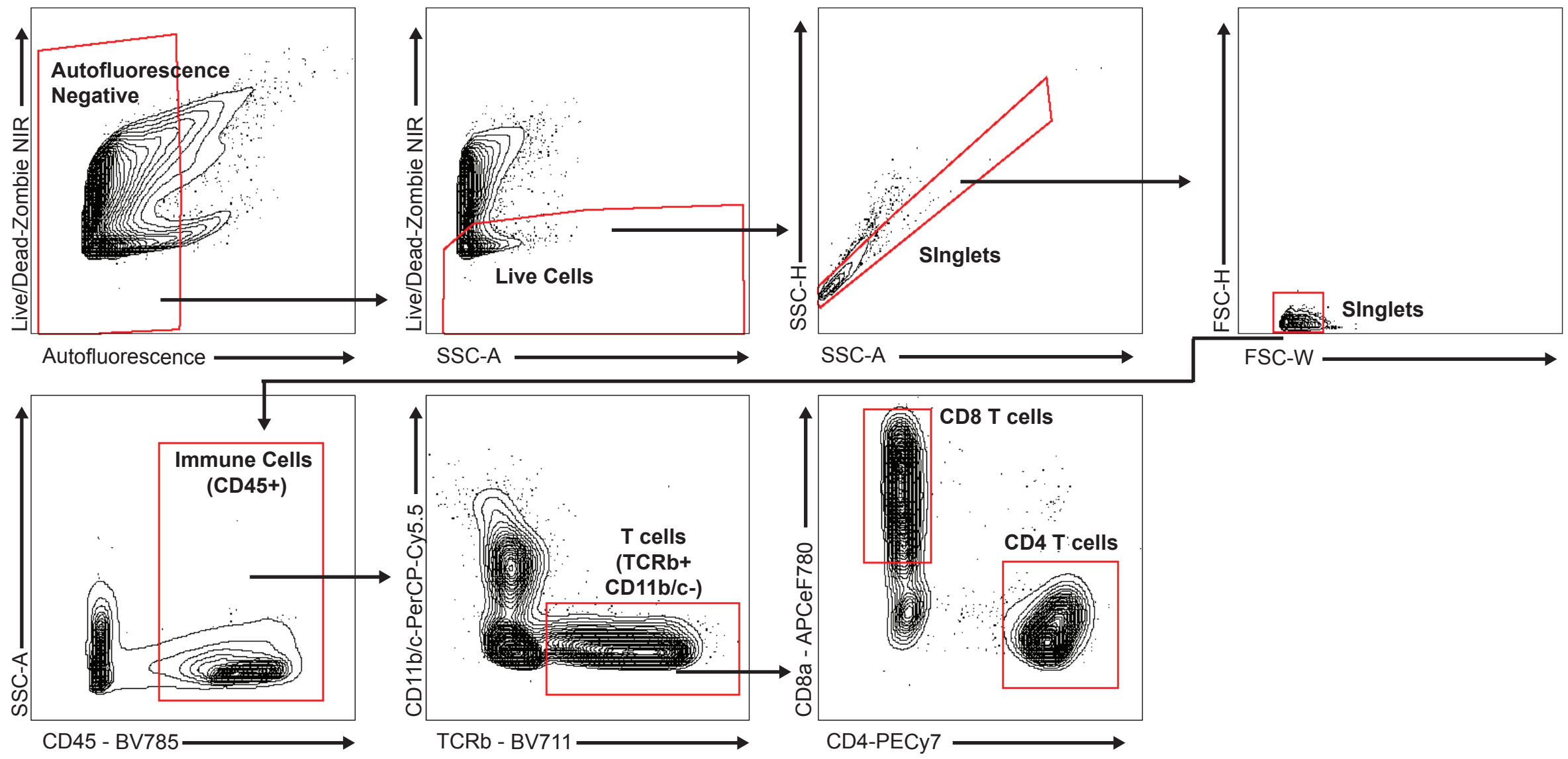

Supplemental Figure 2: Gating Scheme for Flow Cytometry

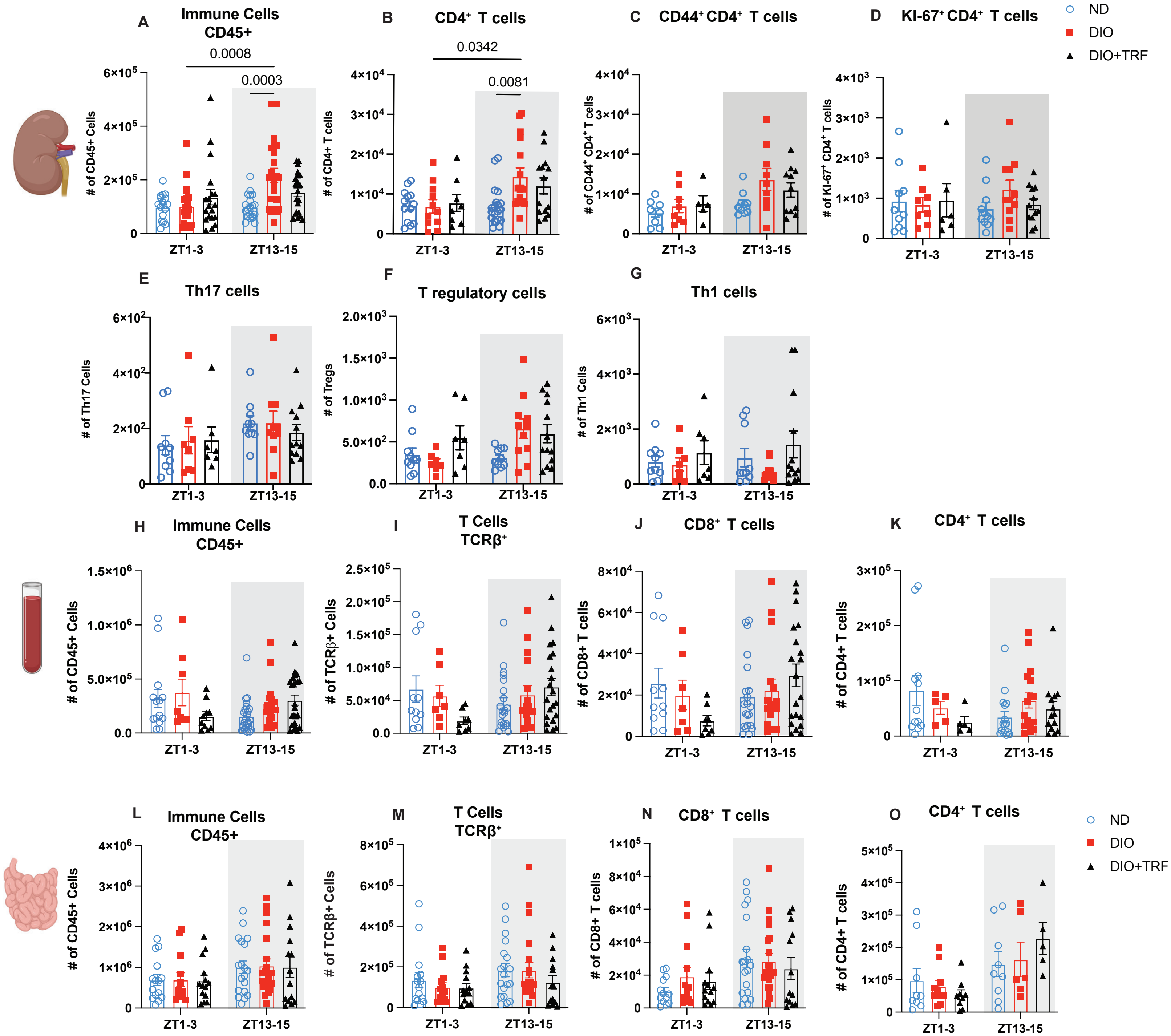

Supplemental Figure 3: Total renal immune cells between ND, DIO and DIO+TRF at ZT1-3 and ZT13-15 (A). Total CD4+ T cells (B), CD44+ CD4+ (C), KI-67+ CD4+ (D), Th17 cells (IL-17A+ CD4+, E), Tregs (FoxP3+ CD4+ F), and Th1 cells (IFNg+ CD4+, G) in ND, DIO, and DIO+TRF mice at ZT1-3 and ZT13-15. Total PBMC counts for immune cells (B), T cells (C), CD8 T cells (D), and CD4 T cells (E) between ND, DIO, and DIO+TRF at ZT1-3 and ZT13-15. Total small intestinal immune cell (F), T cell (G), CD8 T cells (H), and CD4 T cells (I) counts for ND, DIO, and DIO+TRF at ZT1-3 and ZT13-15.

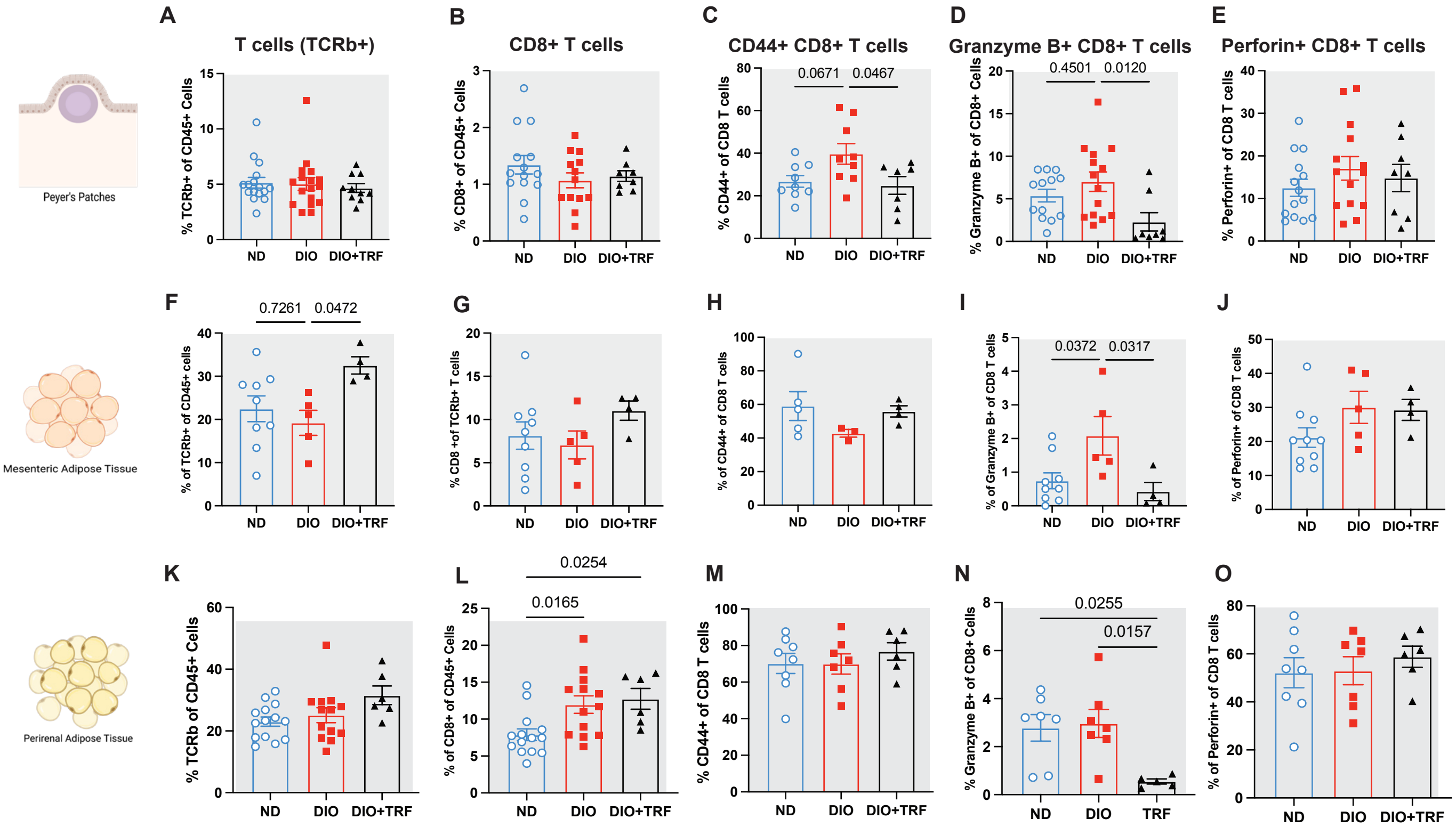

Supplemental Figure 4: At ZT13-15, T cells (A, F, K), CD8 T cells (B, G, L), CD44+ CD8+ T cells (C, H, M), Granzyme B+ CD8+ T cells (D, I, N), and Perforin+ T cells (E, J, O) were analyzed between ND, DIO, and DIO+TRF in the Peyer's patches, mesenteric adipose tissue, and perirenal adipose tissue.

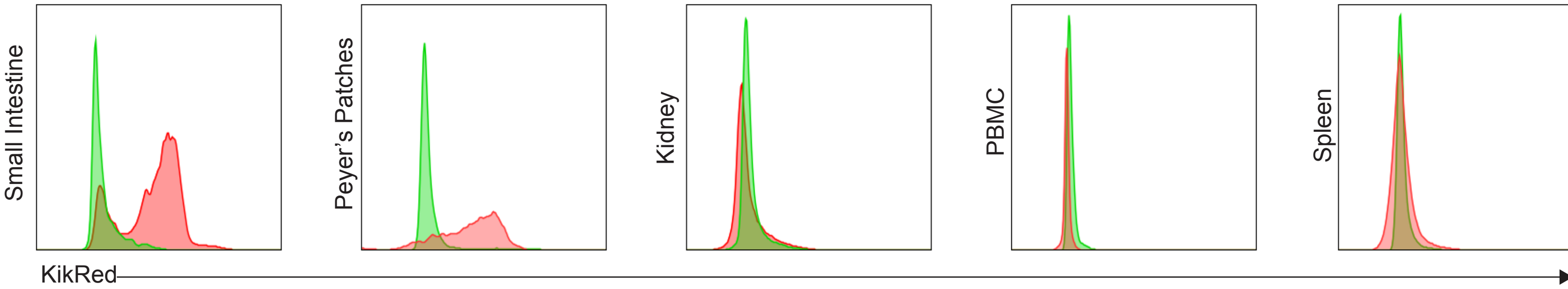

Supplemental Figure 5: KikRed+ signal in Small Intestine, Peyer's patches, Kidney, PBMC, and Spleen directly post photoconversion.

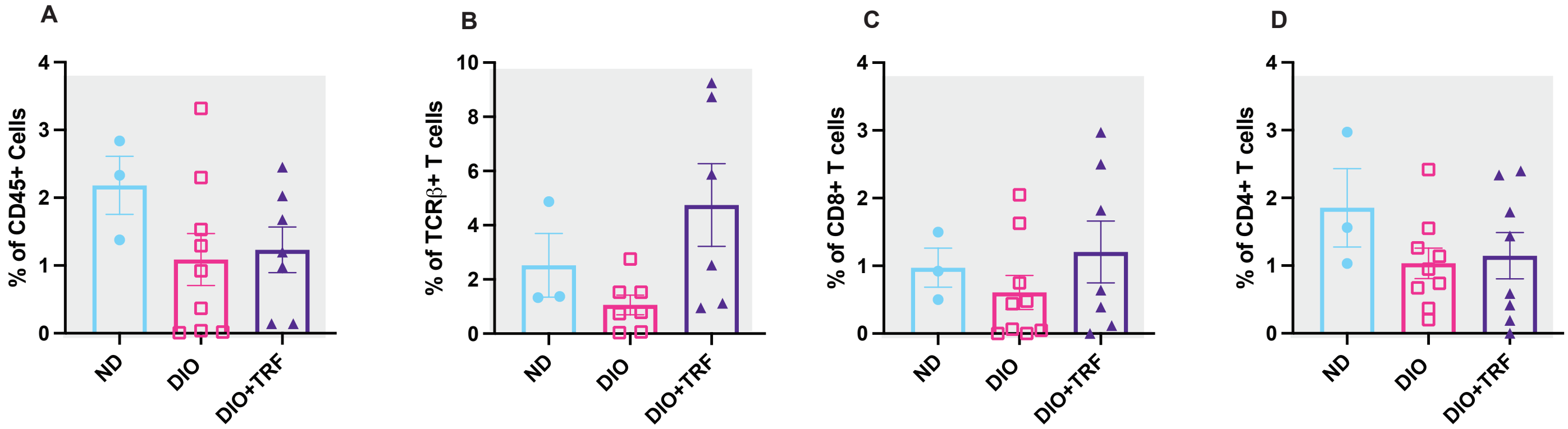

Supplemental Figure 6: PBMC KikRed+ cells of immune cells (A), T cells (B), CD8 T cells (C), and CD4 T cells (D) between ND, DIO, and DIO+TRF.

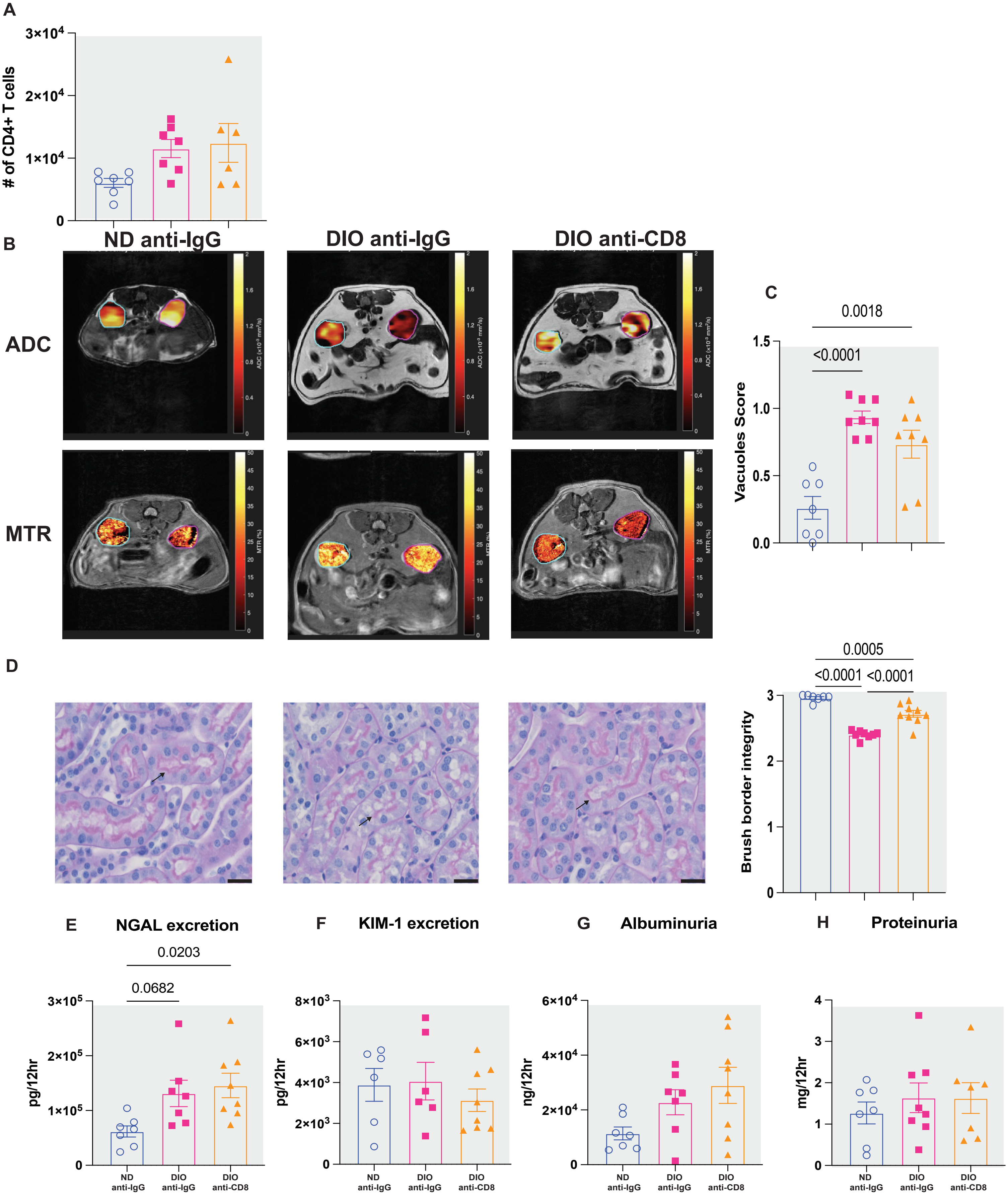

Supplemental Figure 7: CD4+ T cellcounts in the kidney at ZT13 (A). Representative images of MRI for ADC (top) and MTR (bottom) (B). Quantification of tubular vacuoles in ND anti-IgG, DIO anti-IgG, and DIO anti-CD8 (C). Representative 40X images and quantification of proximal tubular brush border integrity in ND anti-IgG, DIO anti-IgG, and DIO anti-CD8 (D). Dark phase urinary NGAL excretion (E), KIM-1 excretion (F), albumin (G), and protein (H) between our 3 groups.

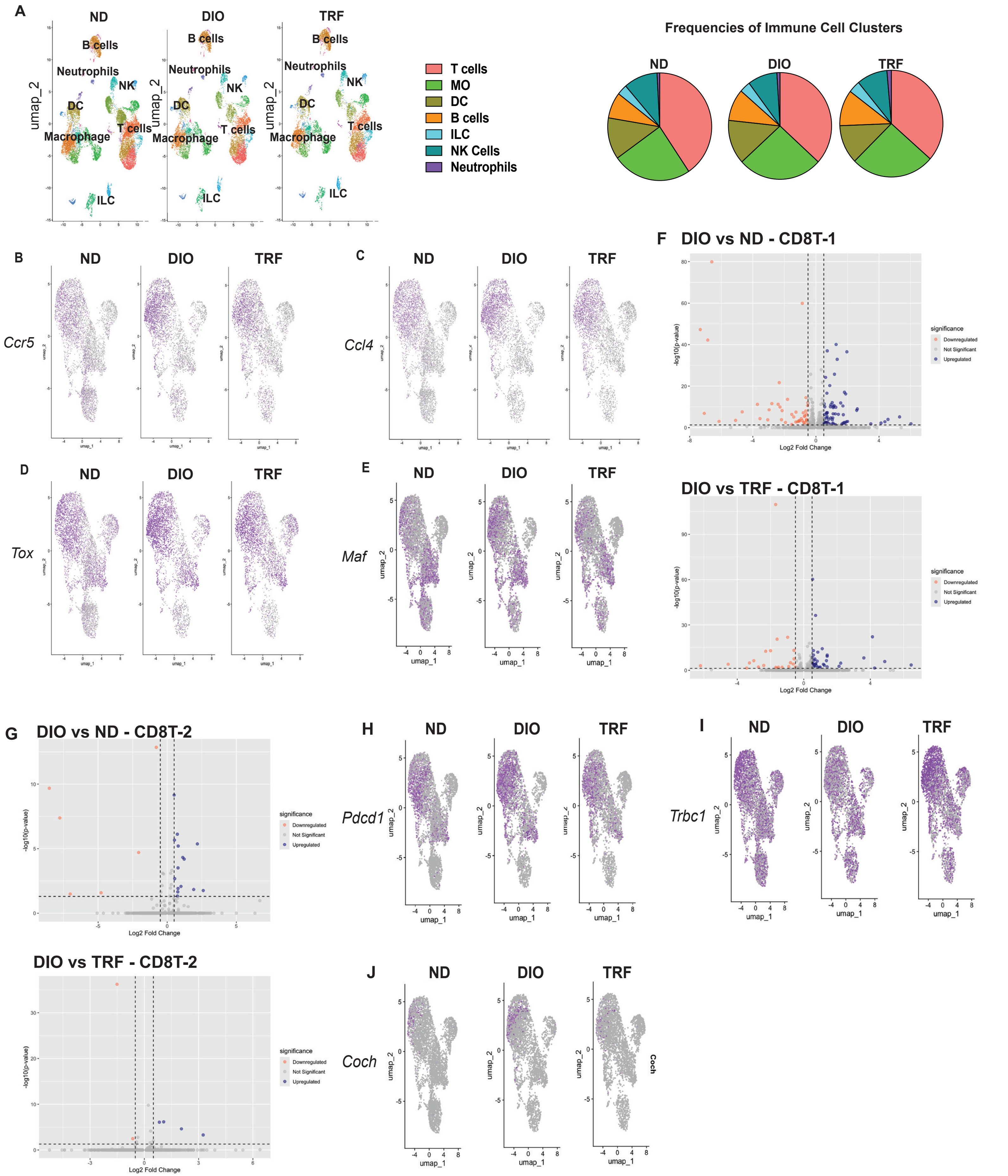

Supplemental Figure 8: UMAP for each immune cell cluster (left) and frequency of all major immune cell populations (right)(A)Feature plots for key for ND, DIO, and DIO+TRF for key CD8T-1 genes, *Ccr5* (A), *Ccl4* (B), *Tox* (C), and *Pdcd1* (D). Volcano plot for all DEGs between DIO vs ND (top) and DIO vs TRF (bottom) in CD8T-1 cluster (E) Volcano plot for all DEGs between DIO vs ND (top) and DIO vs TRF (bottom) in CD8T-2 cluster (F). Feature plots for key for ND, DIO, and DIO+TRF for overlapping DEGs, *Pdcd1* (G), *Trbc1* (H), and *Coch* (I).

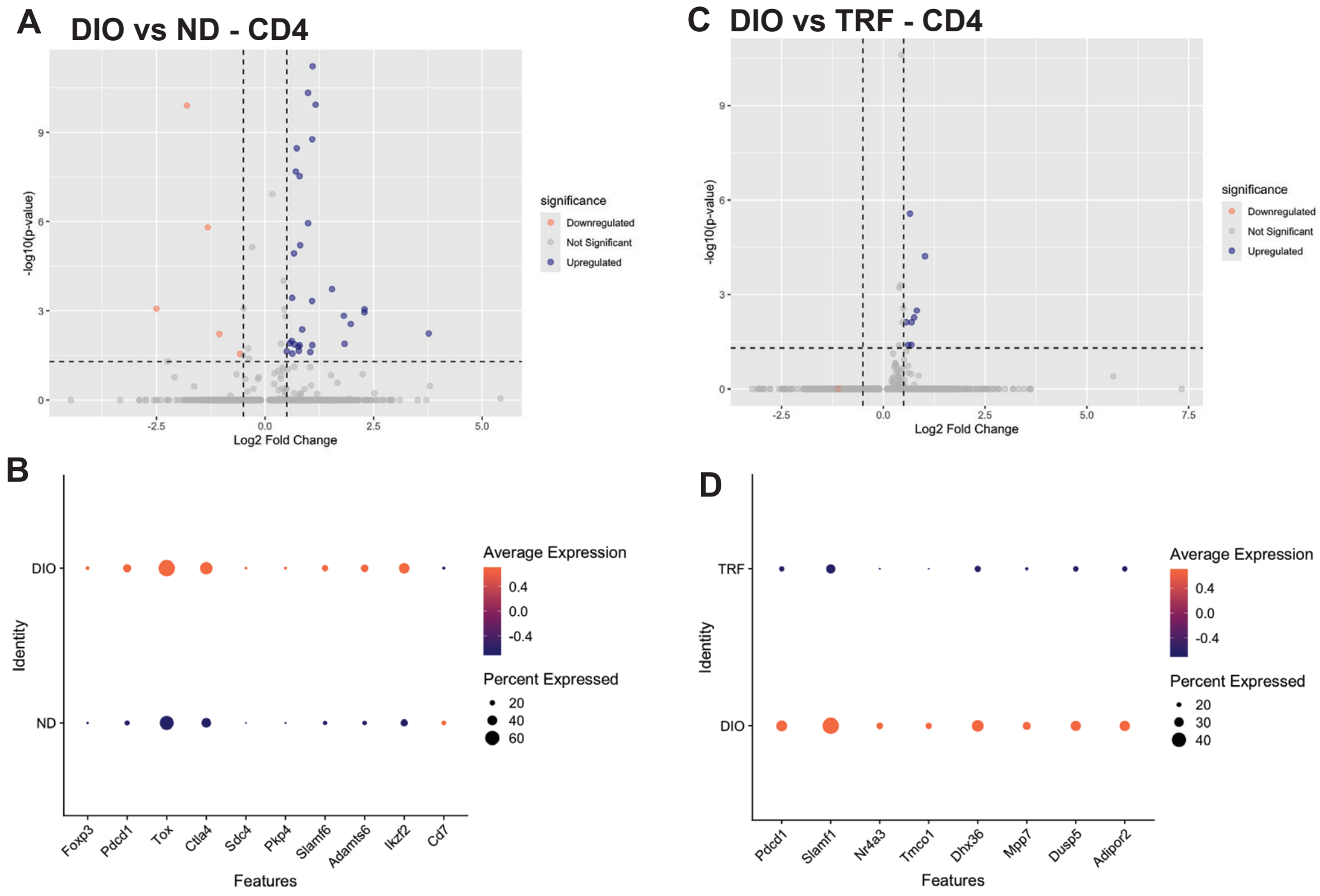

Supplemental Figure 9: Volcano plot for all DEGs between DIO vs ND in CD4 cluster (A). Treg and exhaustion associated DEGs in CD4 between DIO vs ND (B). Volcano plot for all DEGs between DIO vs TRF in CD4 cluster (C). Dot plot of all signifiant DEGs between DIO vs TRF in CD4 (D).

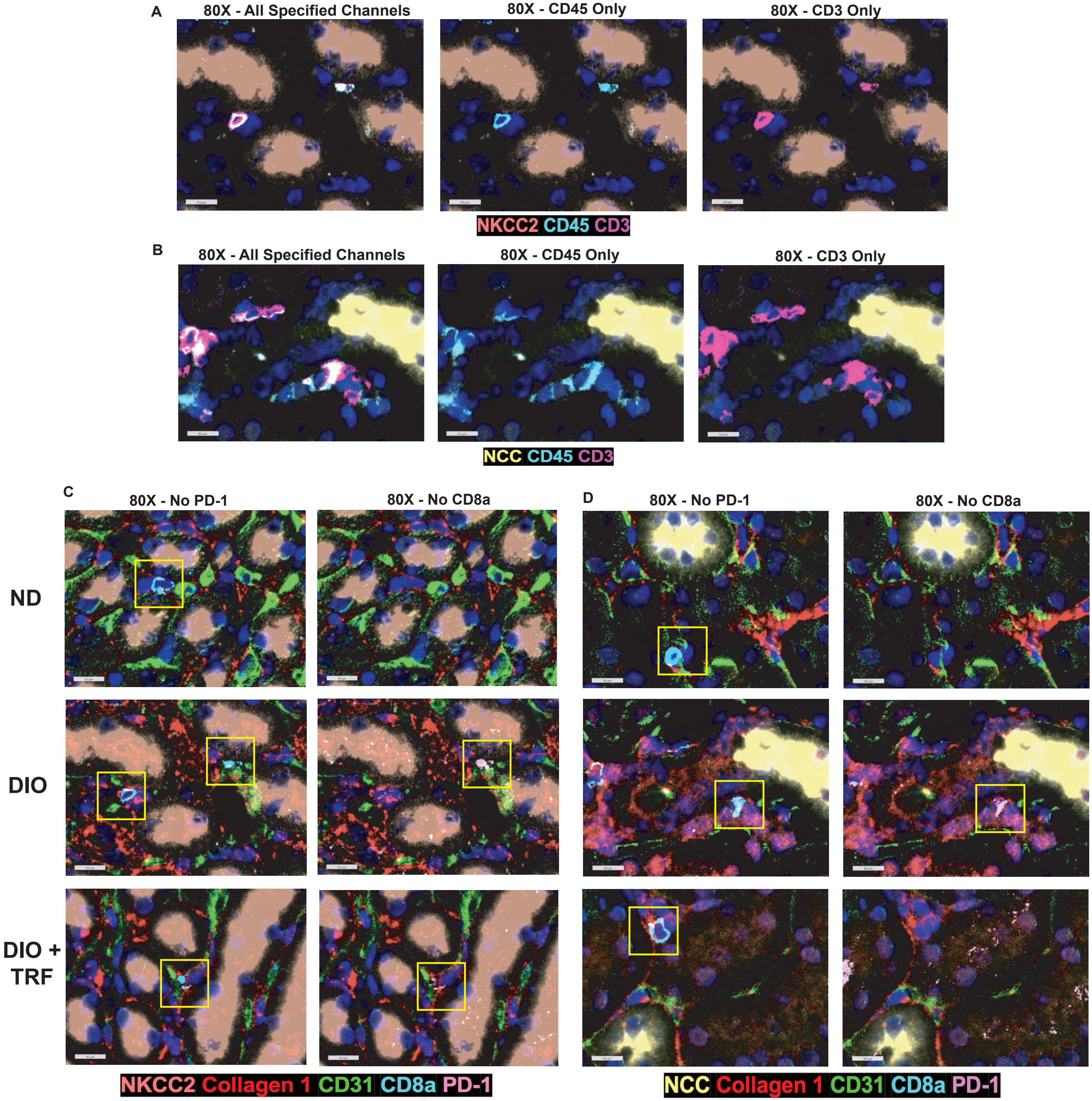

Supplemental Figure 10: Representative 80x images of the kidney medulla (NKCC2, A) and cortex (NCC, B) showing identification of kidney T cells. Representative 80X images of the kidney medulla (NKCC2, C) and kidney cortex (NCC, D) showing identification of endothelial cells (CD31), fibrosis (Collagen 1) and PD-1+ CD8+ T cells in ND, DIO, and DIO+TRF.

Supplemental Table 1:

Gene Name	DIO vs ND Log2FC	DIO vs ND P-value	DIO vs TRF Log2FC	DIO vs ND P-value
Acss2	0.9034	4.4543E-05	1.7721	1.4079E-14
Ddx43	0.5282	0.04319695	0.9151	1.2797E-07
Cd72	1.0736	3.6114E-05	1.4722	1.0713E-06
Trbv2	1.7419	0.00544784	4.9124	8.5299E-07
Ahsa2	0.5515	6.5872E-05	0.7978	7.1972E-06
Maf	0.7301	2.1321E-09	0.7827	1.5294E-11
Cfap74	2.8735	9.7102E-11	3.9246	2.327E-09
Trav16n	3.4582	0.01535436	5.6812	0.0082751
Csf1	2.3493	6.0853E-08	2.1441	1.573E-05
Podnl1	1.3339	2.1419E-09	1.3280	6.6977E-07
Capn11	5.0416	3.7067E-05	6.1969	0.00095459
Hist1h1e	1.1387	1.1551E-11	0.7203	7.3677E-05
Trpc1	1.4188	3.0833E-07	0.9129	0.04713869
Cables1	1.2992	5.2624E-10	0.8759	0.00015512
Il6st	0.6826	0.00063137	0.5412	0.00881356
AC149090.1	0.7298	8.9524E-20	0.6227	3.1333E-15
Camk2b	-3.7180	4.8591E-10	-2.8144	0.00973231
Vmn2r96	2.2282	1.0552E-14	-1.6487	6.7009E-23
Trbc1	-0.9701	7.2137E-73	-1.8308	3.308E-131
Rbm3	-0.5042	5.153E-12	-0.6005	1.7638E-14

Supplemental Table 2:

Gene Name	DIO vs ND Log2FC	DIO vs ND P-value	DIO vs TRF Log2FC	DIO vs ND P-value
Trbc1	-0.6493	1.807606e-09	-1.3043	1.656862e-27
Pdcd1	0.6049	3.369964e-05	0.8007	1.214087e-06
Coch	2.4799	1.296763e-06	2.1184	4.430201e-05
